# Imbalanced Data Correction Based PET/CT Radiomics Model for Predicting Lymph Node Metastasis in Clinical Stage T1 Lung Adenocarcinoma

**DOI:** 10.3389/fonc.2022.788968

**Published:** 2022-01-28

**Authors:** Jieqin Lv, Xiaohui Chen, Xinran Liu, Dongyang Du, Wenbing Lv, Lijun Lu, Hubing Wu

**Affiliations:** ^1^ School of Biomedical Engineering and Guangdong Provincial Key Laboratory of Medical Image Processing, Southern Medical University, Guangzhou, China; ^2^ Guangdong Province Engineering Laboratory for Medical Imaging and Diagnostic Technology, Southern Medical University, Guangzhou, China; ^3^ Nanfang PET Center, Nanfang Hospital, Southern Medical University, Guangzhou, China

**Keywords:** PET/CT, radiomics, lung adenocarcinoma, re-sampling techniques, imbalanced data, lymph node metastasis

## Abstract

**Objectives:**

To develop and validate the imbalanced data correction based PET/CT radiomics model for predicting lymph node metastasis (LNM) in clinical stage T1 lung adenocarcinoma (LUAD).

**Methods:**

A total of 183 patients (148/35 non-metastasis/LNM) with pathologically confirmed LUAD were retrospectively included. The cohorts were divided into training vs. validation cohort in a ratio of 7:3. A total of 487 radiomics features were extracted from PET and CT components separately for radiomics model construction. Four clinical features and seven PET/CT radiological features were extracted for traditional model construction. To balance the distribution of majority (non-metastasis) class and minority (LNM) class, the imbalance-adjustment strategies using ten data re-sampling methods were adopted. Three multivariate models (denoted as Traditional, Radiomics, and Combined) were constructed using multivariable logistic regression analysis, where the combined model incorporated all of the significant clinical, radiological, and radiomics features. One hundred times repeated Monte Carlo cross-validation was used to assess the application order of feature selection and imbalance-adjustment strategies in the machine learning pipeline. Prediction performance of each model was evaluated using the area under the receiver operating characteristic curve (AUC) and Geometric mean score (G-mean).

**Results:**

A total of 2 clinical parameters, 2 radiological features, 3 PET, and 5 CT radiomics features were significantly associated with LNM. The combined model with Edited Nearest Neighbors (ENN) re-sampling methods showed strong prediction performance than traditional model or radiomics model with the AUC of 0.94 (95%CI = 0.86–0.97) vs. 0.89 (95%CI = 0.79–0.93), 0.92 (95%CI = 0.85–0.97), and G-mean of 0.88 vs. 0.82, 0.80 in the training cohort, and the AUC of 0.75 (95%CI = 0.57–0.91) vs. 0.68 (95%CI = 0.36–0.83), 0.71 (95%CI = 0.48–0.83) and G-mean of 0.76 vs. 0.64, 0.51 in the validation cohort. The combination of performing feature selection before data re-sampling obtains a better result than the reverse combination (AUC 0.76 ± 0.06 vs. 0.70 ± 0.07, *p*<0.001).

**Conclusions:**

The combined model (consisting of age, histological type, C/T ratio, MATV, and radiomics signature) integrated with ENN re-sampling methods had strong lymph node metastasis prediction performance for imbalance cohorts in clinical stage T1 LUAD. Radiomics signatures extracted from PET/CT images could provide complementary prediction information compared with traditional model.

## Introduction

Lung cancer is the leading cause of cancer-related deaths in the world, with non-small cell lung cancer (NSCLC) making up 85% of lung cancer cases (1, 2). Lung adenocarcinoma (LUAD) is the most common subtype of NSCLC; the 5-year relative survival rate for LUAD patients diagnosed with regional metastasis and distant metastasis were 44.5 and 8.4% separately, indicating that metastasis is one of the most common fatal causes for LUAD patients (3, 4).

The most common metastatic pathway of clinical T1 stage LUAD is lymphatic metastasis, which determines the treatment strategy and prognosis. Surgical lobectomy combined with systematic lymph node dissection remains the standard therapy for patients with stage T1 LUAD (5, 6). However, there still has a controversy surrounding the idea of whether the systematic lymph node dissection is required for T1 stage LUAD (7, 8). Using the systematic lymph node dissection in early-stage NSCLC without LNM was considered overtreatment (9). A clinical trial demonstrated that compared with mediastinal lymph node sampling, extensive systematic lymph node dissection failed to improve the survival for patients with negative node (10). Further, with the popularity of the “minimally invasive” and “precision medicine” concept, surgeons need to determine the optimal extent of pulmonary resection and lymphadenectomy for individuals (11). Thus, identifying patients with a higher risk of LNM from the stage T1 LUAD will assist surgeons to determine whether the systematic lymph node dissection should be performed or not.

Medical imaging is a non-invasive way to capture tumor phenotypic characteristics. ^18^F-FDG PET/CT combining anatomic data with metabolic information has been applied to guide the staging of NSCLC (12). However, qualitative diagnosis in medical imaging cannot fulfill the clinical demand due to subjective and limited accuracy. Radiomics is an emerging technique that extracts high-throughput data from medical imaging to quantitative tumor phenotypes, using machine learning approaches to construct radiomics signature that can realize the disease prediction and diagnosis (13–15). Our previous studies have demonstrated the great potential of PET/CT radiomics in the diagnosis and prognosis of nasopharyngeal carcinoma, the evaluation of prognosis for head and neck cancer, and differentiation between pulmonary tuberculosis and lung cancer (16–19). Yang et al. (20) developed and validated CT radiomics nomogram to predict LNM in solid lung adenocarcinoma with stage T1–4. Zhong et al. (21) applied CT radiomics signature to predict occult mediastinal lymph node metastasis in clinical T1–3 stage LUAD. Wang et al. (22) utilized peritumoral CT radiomics to predict LNM in patients with stage T1 LUAD. These works focus on single modality CT radiomics to predict LNM in lung adenocarcinoma.

To our knowledge, there has not been any reported prediction of lymph node metastasis in clinical stage T1 lung adenocarcinoma *via* PET/CT radiomics. We hypothesize that using radiomics analysis based PET/CT can identify LUAD patients with a high risk of lymph node metastasis. In present study, the imbalance-adjustment strategies using ten data re-sampling methods were adopted to balance the distribution of majority (non-metastasis) class and minority (lymph node metastasis) class. Then, three multivariate models (denoted as Traditional, Radiomics, and Combined) were constructed using multivariable logistic regression analysis, where the combined model incorporated all of the significant clinical, radiological, and radiomics features. Prediction performance of each model was evaluated using the area under the receiver operating characteristic curve (AUC) and Geometric mean score (G-mean).

## Materials and Methods

### Patients

**Figure 1:**
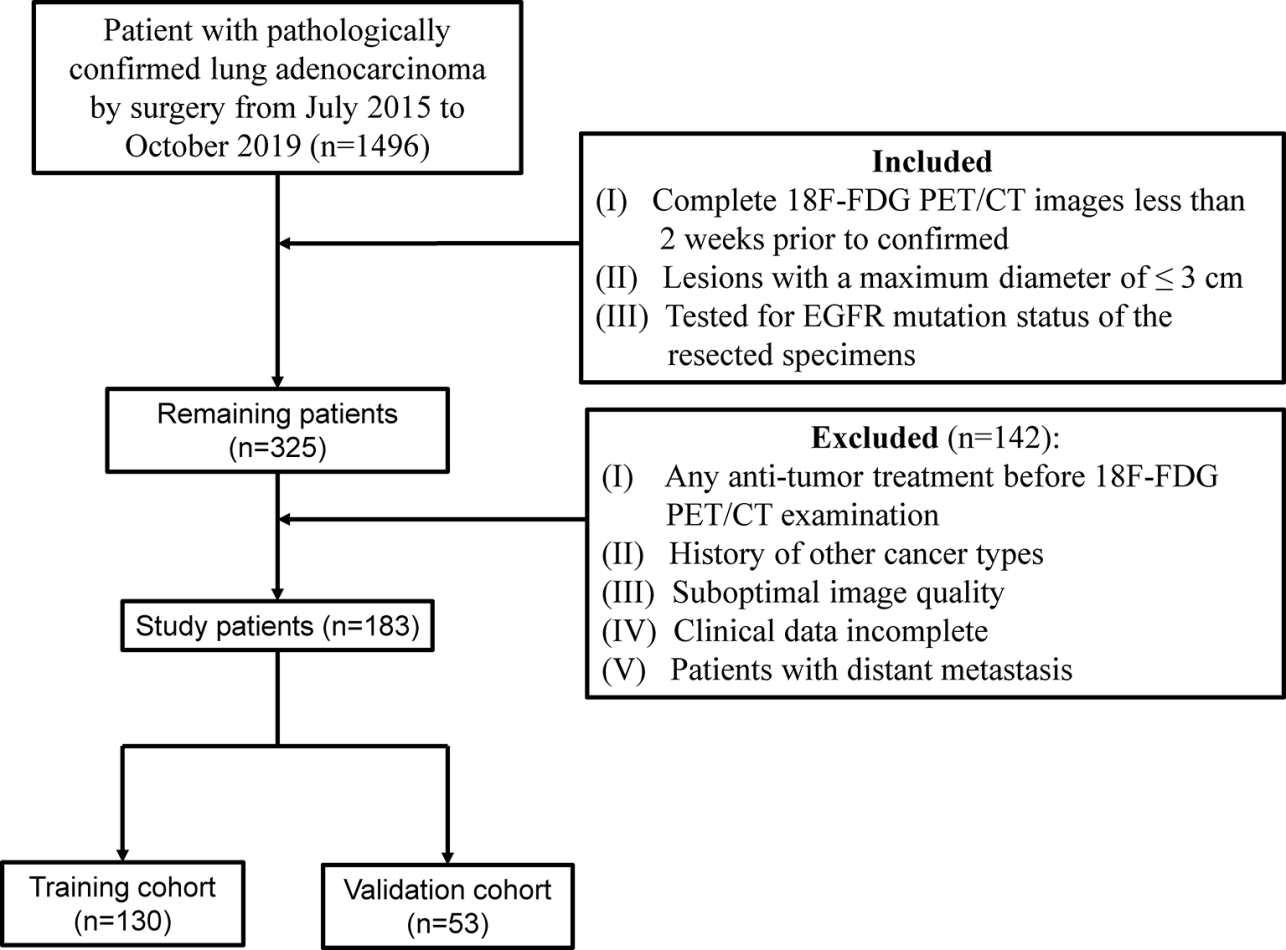
Flow chart of the study of the enrolled patients.

This retrospective study was approved by the Institutional Review Boards, and the need for informed consent for patients was waived. According to the inclusion and exclusion criteria (**Figure 1**), a total of 183 patients who had been surgically treated for ≤3 cm clinical stage T1 LUAD were enrolled and randomly allocated to the training and validation cohort at a ratio of 7:3. Additional information on LNM evaluation and histopathologic classification criterion is available in **Supplementary Method S1**.

### PET/CT Image Acquisition, Segmentation, Radiological and Radiomics Feature Extraction

**Figure 2:**
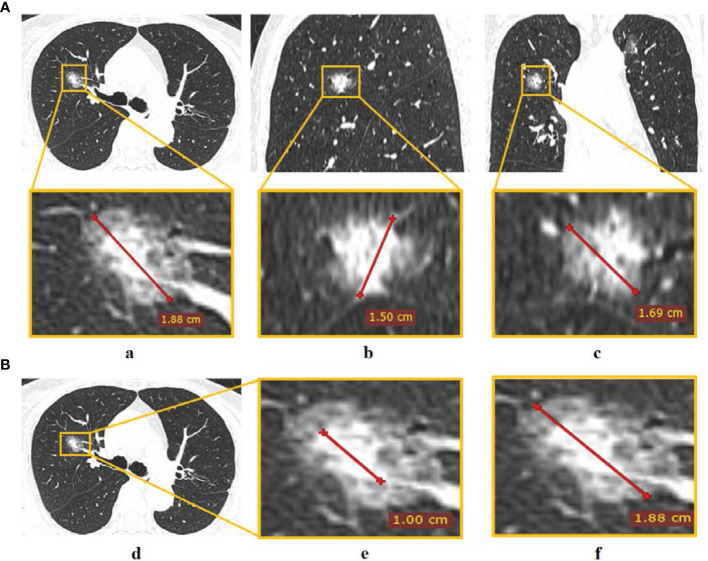
The measurement of CT radiological features. **(A)** Tumor diameter measured in (a) transverse, (b) sagittal, and (c) coronal planes, and the largest diameter in the three planes was defined as MTD; **(B)** C/T ratio measurement of (d) tumor *via* (e) maximum consolidation diameter divided by (f) maximum tumor diameter in the transverse plane.

Two CT radiological features, namely, maximum tumor diameter (MTD) and the consolidation-to-tumor (C/T) ratio were extracted. MTD was defined as the largest tumor diameter among three planes (transverse, sagittal, and coronal) (**Figure 2A**) (23). C/T ratio was defined as the maximum consolidation (C) diameter divided by the maximum tumor (T) diameter in the transverse plane (**Figure 2B**). The details of PET/CT image acquisition, tumor segmentation, MTD and C/T ratio measurement, radiomics feature extraction, and inter-observer reproducibility analysis can be found in the **Supplementary Information**. The inter-class correlation coefficient (ICC) was used to evaluate the inter-observer reproducibility of MTD, C/T ratio, and radiomics features.

### Re-Sampling Methods for Unbalance Correction

Due to the highly unbalanced ratio between non-metastasis and lymph node metastasis cases (148:35 in this study), models maybe inclined to make a prediction of majority class during training process. To alleviate this negative effect, we use several re-sampling techniques to balance the distribution in training cohort for model construction. The re-sampling techniques should be applied only to training data to avoid data leakage, while the validation cohort was untouched as it represents the real situation in clinical practice (24).Ten re-sampling techniques worked in our study: random over-sampling (ROS), Adaptive Synthetic (ADASYN), Synthetic Minority Oversampling Technique (SMOTE), Borderline SMOTE (bSMOTE), Random under-sampling (RUS), NearMiss (NM), Tomek links (TL), Edited Nearest Neighbours (ENN), Over-sampling using SMOTE and cleaning using Tomek links (SMOTE-TL) and Over-sampling using SMOTE and cleaning using ENN (SMOTE-ENN) (25–27). The re-sampling methods are described in the **Supplementary Method S7**.

### Radiomics Feature Selection and Model Construction

**Figure 3:**
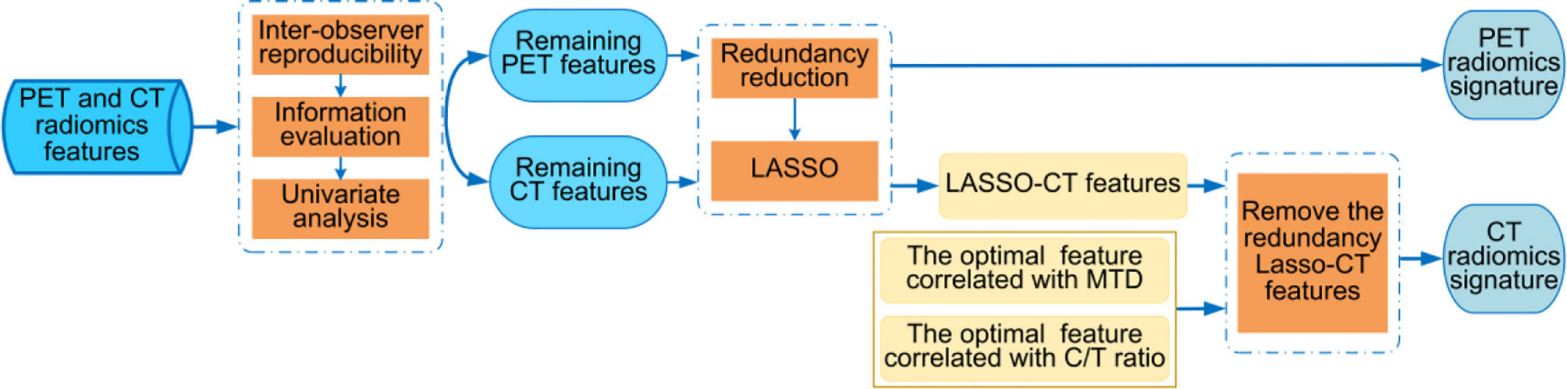
The workflow of radiomics features selection.

The process of feature selection consisted of the following three parts. Part 1, through the inter-observer reproducibility, information evaluation, and univariate analysis, the robust and useful features were selected as the remaining PET or CT features from the PET and CT radiomics features. Part 2, PET radiomics feature selection. After the redundancy reduction, the least absolute shrinkage and selection operator (LASSO) penalty logistic regression is used to determine the PET radiomics signature from the remaining PET features. Part 3, CT radiomics feature selection. The LASSO-CT features were selected from the remaining CT features *via* the methods same with part 2. MTD and C/T ratio were prognostic factors for lung adenocarcinoma (28, 29), however, due to their high inter-observer variability, we want to select two optimal correlated radiomics features with high robustness to alternative these radiological characteristics. The optimal correlated radiomics features were selected from the CT morphological features and the CT intensity-based statistical features separately *via* Pearson correlation analysis and 50 times 5-fold cross-validation. Next, we removed the redundancy LASSO-CT features that interactive with the two optimal correlated radiomics features, the remainder features were considered as the CT radiomics signature. The processes of radiomics feature selection are shown in **Figure 3** and more details are described in **Supplementary Method S8**. The feature selection process was performed on the training cohort and validated in the validation cohort.

PET/CT radiomics signature was generated by combing two signatures of CT and PET images. The PET/CT radiomics model was developed by the multivariable logistic regression. The Rad-score of each patient was calculated using a linear combination of selected radiomics feature weighted by their respective coefficients.

### Development of the Traditional Model and Combined Model

**Figure 4:**
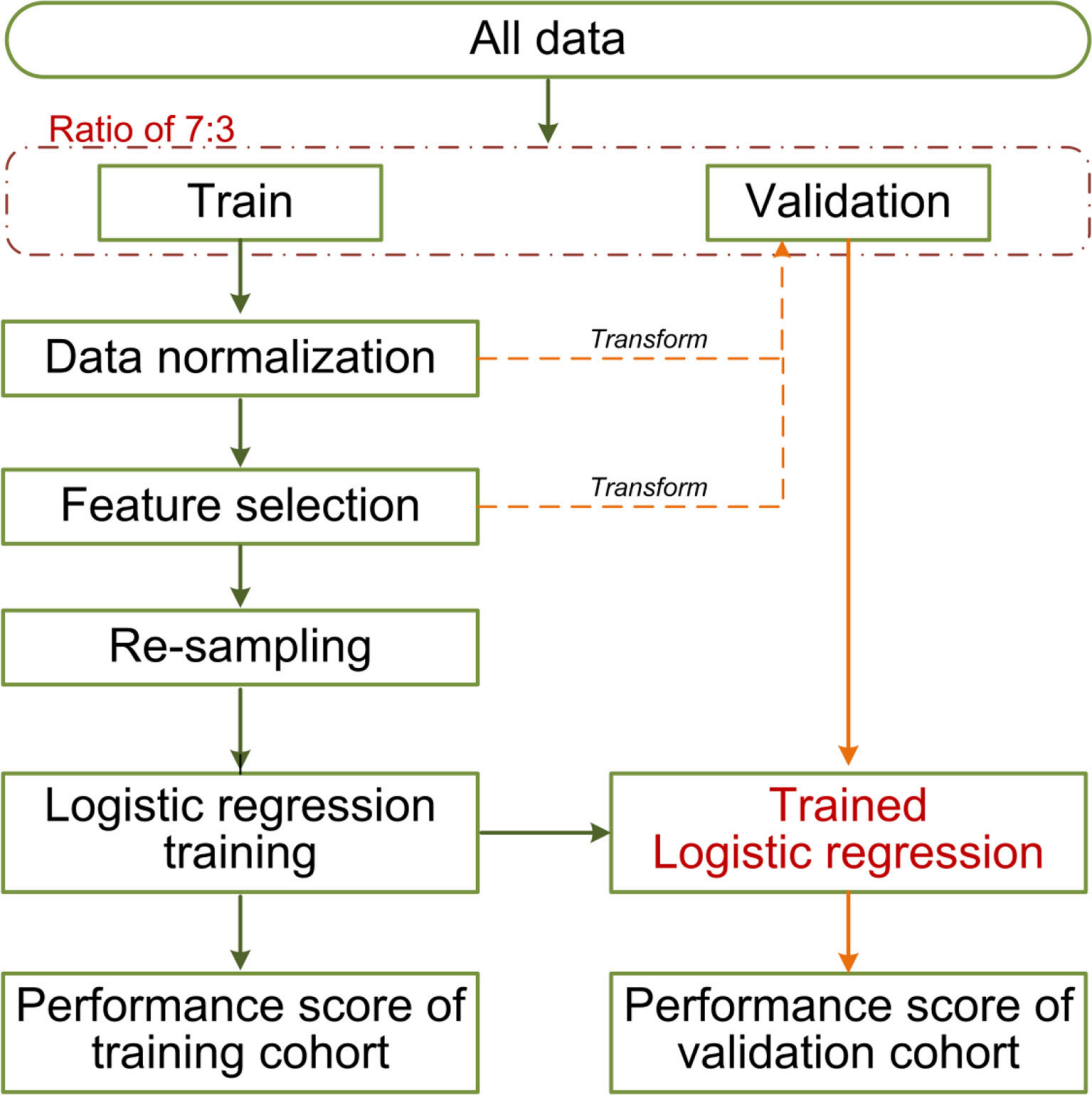
The machine learning pipeline.

The clinicopathological/radiological features included gender, age, histologic subtype, EGFR mutation, MTD, C/T ratio, MATV, TLG, SUVpeak, SUVmean, and SUVmax. Univariate logistic regression analysis was used to assess the association between clinicopathological/radiological features and LUAD lymph node metastasis in the training cohort, only the significant features with a *p* <0.05 were put into multivariable logistic regression (employing backward step-wise elimination with the Akaike information criterion as the stopping rule) for traditional model construction. A combined model incorporating the selected clinicopathological/radiological features, and Rad-score was established. The machine learning pipelines are shown in **Figure 4**.

### Model Comparison and Evaluation

AUC was used to evaluate the prediction performance, and comparison between AUCs was conducted by Delong’s test (30). The accuracy (ACC) will bias the result towards the majority class, especially in the class imbalanced data. The Geometric mean score (G-mean), which is defined as the geometry mean of accuracy (also the arithmetic mean of sensitivity and specificity) (31) can balance the accuracy of the majority and minority class, thus, we used G-mean instead of ACC.

Precision of majority class (maP) was a fraction of correctly predicted non-metastasis samples among the ones labeled as non-metastasis samples. Recall of majority class (maR) was the fraction of non-metastasis instances that have been retrieved over the total number of non-metastasis instances. The relevant cases were LNM samples for the precision of minority class (miP) and recall of minority class (miR). F-measure of majority/minority class (maF/miF) was a harmonic mean between recall and precision, which considered them as similarly crucial. For all these metrics, larger values indicated better performance. Evaluation metric formulas are listed in **Supplementary Method S9**.

### Statistical Analysis

The subsequent result demonstrated that it is difficult to predict lymph node metastasis for the solid tumors in the LUAD. Thus, we subdivided 92 patients into the solid-tumor subgroup by the C/T ratio was one for further analysis (32).

**Figure 5:**
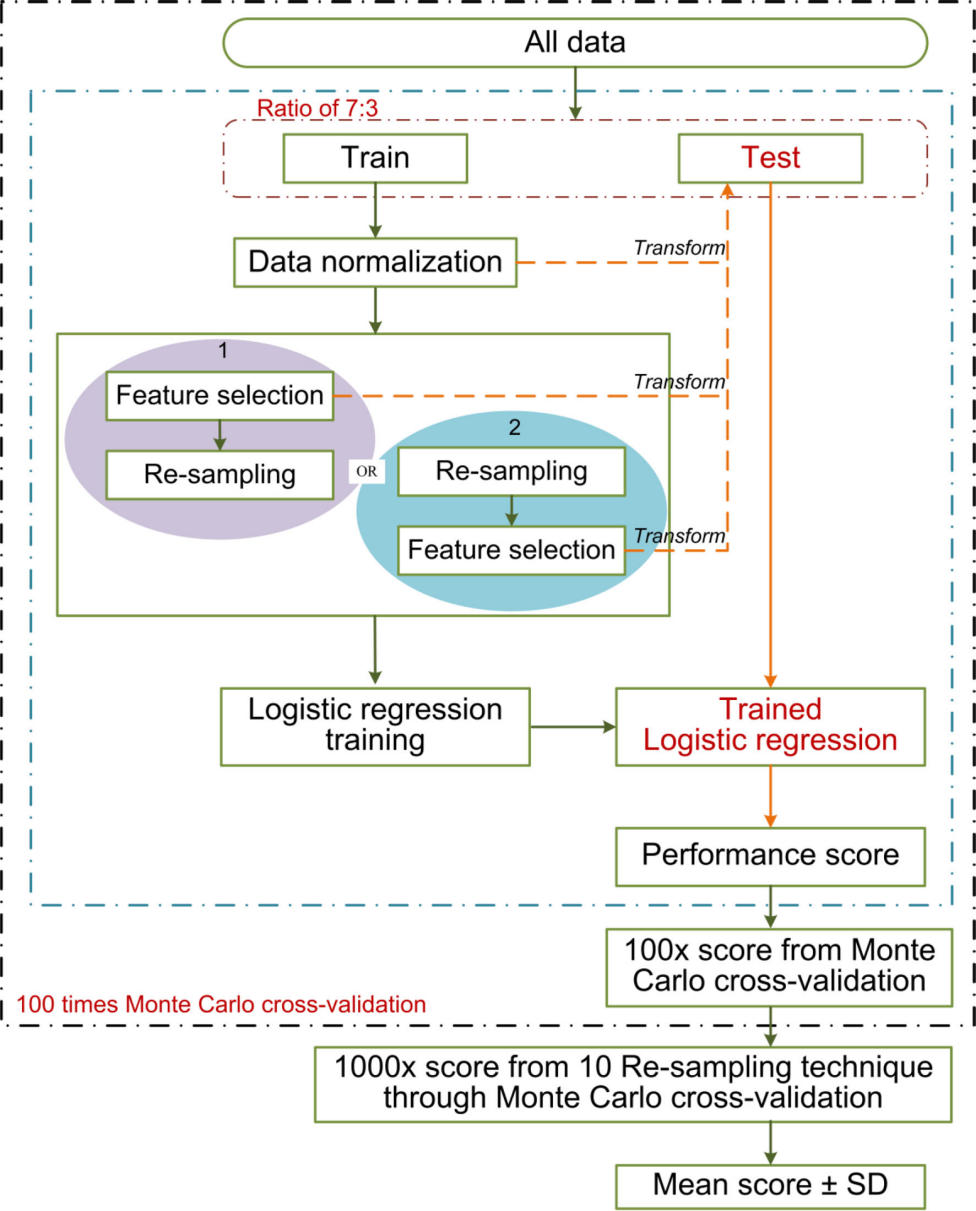
The schematic overview of the 100 times repeated Monte Carlo cross-validation for the two different sequences. The cross-validation randomly divides all data 100 times into training and testing cohorts at a ratio of 7:3. The two different sequences are shown in red and blue separately. The data normalization, the implemented sequences, and the model training were performed within each training cohort (100 training cohorts in total). Each corresponding test cohort yields a performance score. This process can prevent leakage of test data into the trained model. We applied 10 re-sampling methods, each re-sampling method has 100 performance scores from the cross-validation. The mean score of the specified sequences was calculated from the average of 1,000 performance scores (10 re-sampling methods × 100 times cross-validation of per re-sampling method).

Approximately 100 times of Monte Carlo cross-validation were implemented to randomly divide all data into training and testing cohorts at a ratio of 7:3 to obtain the average results for further statistical analysis. The statistical difference was analyzed by Mann–Whitney U test. In the machine learning pipeline, we performed feature selection before data re-sampling. To prove this application order was appropriate for the present study, two different sequences were designed for the statistical analysis: (1) feature selection was performed before re-sampling data; (2) feature selection was performed after re-sampling data (feature was selected from the robust features). The schematic overview of the 100 times repeated Monte Carlo cross-validation for the two different sequences is shown in **Figure 5**. On the other, the statistical analysis was applied in comparing the predictive performance between MTD or C/T ratio and their related optimal radiomics features individually.

All of the statistical analyses were performed using MATLAB R2018b, Statistical Program for Social Science (SPSS; version 22.0), and R software (version 4.0.3; http://www.Rproject.org). A two-tailed test p-value <0.05 was considered statistically significant. Re-sampling techniques were completed by Python (version 3.7.0) machine learning library named imbalanced-learn (version 0.5.0) with default settings (33).

## Results

### Patient Characteristics

A total of 183 patients (148/35 non-metastasis/LNM) were enrolled in our study, the imbalance ratio was 1:4. Patient characteristics are described in **Table 1**. No significant differences were found in the overall stage, patient number of LNM, clinicopathological features, and radiological features between the training and validation cohort. The imbalance rate after re-sampling in the training cohort is listed in **Supplementary Table S1**.

**Table 1:** Patient characteristics.

Characteristic	Overall	Training	Validation	*p-*value
	N = 183	N = 130 (70.0%)	N = 53 (30.0%)	
Age (years)	58.05 ± 9.53	59.95 ± 8.63	56.83 ± 11.24	0.08
Gender				0.56
Male	85 (46.5%)	58 (44.6%)	27 (50.9%)	
Female	98 (53.5%)	72 (55.4%)	26 (49.1%)	
Overall stage				0.51
IA1	1 (0.5%)	0 (0%)	1 (1.9%)	
IA2	83 (45.4%)	63 (48.5%)	20 (37.7%)	
IA3	64 (35.0%)	41 (31.5%)	23 (43.4%)	
IIB	11 (6.0%)	8 (6.1%)	3 (5.7%)	
IIIA	23 (12.6%)	17 (13.1%)	6 (11.3%)	
IIIB	1 (0.5%)	1 (0.8%)	0 (0%)	
Histologic subtype				0.76
AIS	10 (5.5%)	8 (6.2%)	2 (3.8%)	
MIA	14 (7.7%)	7 (5.4%)	7 (13.2%)	
LPA	61 (33.3%)	44 (33.8%)	17 (32.1%)	
APA	59 (32.2%)	42 (32.3%)	17 (32.1%)	
PPA	13 (7.1%)	11 (8.5%)	2 (3.8%)	
SPA	18 (9.8%)	12 (9.2%)	6 (11.2%)	
IMA	8 (4.4%)	6 (4.6%)	2 (3.8%)	
EGFR mutation				0.83
Yes	113 (61.8%)	80 (61.5%)	33 (62.3%)	
No	70 (38.2%)	50 (38.5%)	20 (37.7%)	
MTD (cm)	2.05 ± 0.54	2.06 ± 0.52	2.04 ± 0.59	0.81
C/T ratio	0.72 ± 0.33	0.73 ± 0.33	0.69 ± 0.34	0.53
MATV (cm^3^)	8.18 ± 6.03	8.06 ± 5.65	8.47 ± 6.92	0.93
TLG	21.75 ± 25.36	20.91 ± 22.64	23.76 ± 31.21	0.86
SUVpeak	4.42 ± 3.32	4.37 ± 3.23	4.56 ± 3.57	0.89
SUVmean	2.10 ± 1.15	2.08 ± 1.12	2.14 ± 1.24	0.95
SUVmax	7.21 ± 5.42	7.09 ± 5.25	7.441 ± 5.84	0.90
LNM				0.64
Yes	35 (19.1%)	26 (20%)	9 (17%)	
No	148 (80.9%)	104 (80%)	44 (83%)	

Categorical variables are in N (%) and analyzed using chi-squared, while continuous variables are in mean ± SD and analyzed using Student’s t-test or the Mann–Whitney U test, as appropriate. LNM, Lymph node metastasis; AIS, Adenocarcinoma in situ; MIA, Minimally invasive adenocarcinomas; LPA, Lepidic predominant invasive adenocarcinomas; APA, Acinar predominant adenocarcinomas; PPA, Papillary predominant adenocarcinomas; SPA, Solid predominant invasive adenocarcinomas; IMA, Invasive mucinous adenocarcinomas; MTD, Maximum tumor diameters; C/T ratio, consolidation-to-tumor; MATV, metabolically active tumor volume; TLG, total lesion glycolysis. *p < 0.05 indicates the significant difference.

### Construction and Performance of Radiomics Model

A total of 487 PET radiomics features and 487 CT radiomics features were extracted. After the feature selection process of Part 1, 368 and 272 radiomics features were included for PET and CT remaining features separately. Next, 3 PET radiomics features were eventually determined in Part 2. In Part 3, 5 CT radiomics features were ultimately selected, namely, morph_av (the optimal feature correlated with MTD), stat_median (the optimal feature correlated with C/T ratio), and 3 LASSO-CT features. The details of feature selection and computation of Rad-score are presented in **Supplementary Results S1, S2**.

Radiomics model derived the AUC of 0.86 (95%CI: 0.77–0.91) and G-mean of 0.80 in the training cohort, and AUC of 0.67 (95%CI: 0.51–0.85) and G-mean of 0.54 in the validation cohort.

### Development and Validation of the Traditional Model and Combined Model

Age, histologic subtype, and all radiological features were significantly associated with LNM in univariate logistic regression analysis (**Supplementary Table S2**). Age, histologic subtype, C/T ratio, and MATV were finally identified after backward step-wise elimination, and those predictors were used to establish the traditional model. A combined model incorporating the above predictors and Rad-score was constructed.

Performances of the proposed models are presented in **Table 2**. The traditional model achieved the AUC of 0.84 (95%CI, 0.78–0.89) and G-mean of 0.80 in the training cohort, and an AUC of 0.67 (95%CI, 0.38–0.83) and G-mean of 0.65 in the validation cohort. The prediction performance of the traditional model was almost equal to the radiomics model. The combined model outperformed traditional model in both the training [AUC, 0.88 (95%CI: 0.82–0.93); *p* = 0.19] and validation cohorts [AUC, 0.70 (95%CI: 0.52–0.84); *p* = 0.51], without statistically significant.

**Table 2:** Predictive performance for the proposed models.

Models	Training cohort (n = 130)								Validation cohort (n = 53)							
	AUC	G-mean	maP	maR	maF	miP	miR	miF	AUC	G-mean	maP	maR	maF	miP	miR	miF
	(95%CI)								(95%CI)							
Radiomics	0.86	0.80	0.95	0.76	0.84	0.47	0.85	0.60	0.67	0.54	0.85	0.66	0.74	0.21	0.44	0.29
	(0.77–0.91)								(0.51–0.85)							
Traditional	0.84	0.80	0.94	0.79	0.86	0.49	0.81	0.61	0.67	0.65	0.89	0.75	0.81	0.31	0.56	0.40
	(0.78–0.89)								(0.38–0.83)							
**Combined**	**0.88**	**0.83**	**0.95**	**0.86**	**0.90**	**0.58**	**0.81**	**0.68**	**0.70**	**0.43**	**0.84**	**0.84**	**0.84**	**0.22**	**0.22**	**0.22**
	**(0.82–0.93)**								**(0.52–0.84)**							

AUC, Area under the receiver operating characteristic; G-mean, Geometric mean score; maP, Precision of majority class; maR, Recall of majority class; maF, F-measure of majority class; miP, Precision of minority class; miR, Recall of minority class; miF, F-measure of minority class. The model with the best predictive performance was in bold.

### Effectiveness of Re-Sampling Methods on Performance of Models

**Figure 6:**
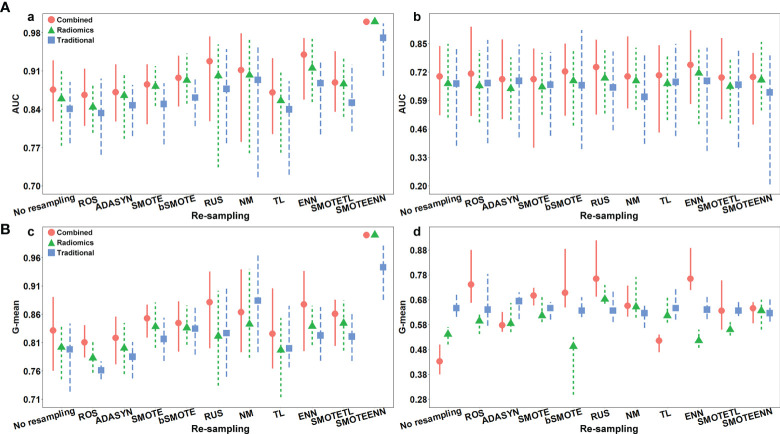
**(A)** The AUC with the confidence intervals of the proposed models with and without re-sampling techniques in (a) training and (b) validation cohorts; **(B)** the G-mean with the confidence intervals of proposed models with and without re-sampling techniques in (c) training and (d) validation cohorts.

The AUC and G-mean of proposed models with and without re-sampling techniques are presented in **Figures 6A, B** separately. The performance of each model combing with re-sampling techniques and the effective re-resampling techniques are described in **Supplementary Result S3**. Overall, the ENN re-sampling technique supported the proposed models to generate satisfactory prediction performance (**Table 3**). Radiomics model combined with ENN achieved improvement performance compare to no re-sampling in the validation cohort [AUC, 0.71 (95%CI, 0.48–0.83) *vs.* 0.67 (95%CI: 0.51–0.85), *p* = 0.71)]. Traditional model combined with ENN obtained better performance than no re-sampling in validation cohort [AUC, 0.68 (95%CI: 0.36–0.83) *vs.* 0.67 (95%CI: 0.38–0.83), *p* = 0.59)]. Combined model trained with ENN did not make a statistically significant improvement performance compare to no re-sampling in the validation cohort [AUC, 0.75 (95%CI: 0.57–0.91) *vs.* 0.70 (95%CI: 0.52–0.84), *p* = 0.06)].

**Table 3:** Predictive performance for combination of ENN re-sampling method in the proposed models.

ENN+ Models	Training cohort (n = 130)								Validation cohort (n = 53)							
	AUC	G-mean	maP	maR	maF	miP	miR	miF	AUC	G-mean	maP	maR	maF	miP	miR	miF
	(95%CI)								(95%CI)							
ENN+ Radiomics	0.92	0.80	0.95	0.75	0.84	0.48	0.85	0.61	0.71	0.51	0.85	0.80	0.82	0.25	0.33	0.29
	(0.85–0.97)								(0.48–0.83)							
ENN+ Traditional	0.89	0.82	0.97	0.70	0.82	0.61	0.96	0.75	0.68	0.64	0.90	0.61	0.73	0.26	0.67	0.38
	(0.79–0.93)								(0.36–0.83)							
**ENN+ Combined**	**0.94**	**0.88**	**0.94**	**0.87**	**0.90**	**0.77**	**0.88**	**0.82**	**0.75**	**0.76**	**0.94**	**0.75**	**0.84**	**0.39**	**0.78**	**0.52**
	**(0.86–0.97)**								**(0.57–0.91)**							

ENN, Edited Nearest Neighbors; AUC, Area under the receiver operating characteristic; G-mean, Geometric mean score; maP, Precision of majority class; maR, Recall of majority class; maF, F-measure of majority class; miP, Precision of minority class; miR, Recall of minority class; miF, F-measure of minority class. The model with the best predictive performance was in bold.

### Radiomics Model Demonstrated Brilliant Predictive Power for Solid Tumor Metastasis

The patient characteristics in the solid tumor subgroup were illustrated in **Supplementary Table S3**. The predictive performance of the proposed models in the solid tumor subgroup is shown in **Table 4**. The radiomics model displayed excellent performance with the AUC of 0.75 (95%CI: 0.61–0.85) and 0.59 (95%CI: 0.34–0.90) in the training and validation cohorts separately. The traditional model showed a relatively low AUC of 0.69 (95%CI: 0.54–0.83) and 0.54 (95%CI: 0.32–0.78) in the training and validation cohort, respectively. The combined model outperformed traditional model in the training cohort [AUC, 0.79 (95%CI: 0.61–0.88); *p* = 0.05], while slightly worse performance in the validation cohorts probably due to the limited number of validation samples [AUC, 0.53 (95%CI: 0.25–0.78); *p* = 0.91]. (ROC curves of the proposed models in the solid tumor subgroup are shown in **Supplementary Figure S1**).

**Table 4:** Predictive performance for the proposed models in the solid-tumor subgroup.

Models	Training cohort (n = 65)								Validation cohort (n = 27)							
	AUC	G-mean	maP	maR	maF	miP	miR	miF	AUC	G-mean	maP	maR	maF	miP	miR	miF
	(95%CI)								(95%CI)							
**Radiomics**	**0.75**	**0.74**	**0.90**	**0.63**	**0.74**	**0.54**	**0.86**	**0.67**	**0.59**	**0.55**	**0.78**	**0.39**	**0.52**	**0.78**	**0.83**	**0.52**
	**(0.61–0.85)**								**(0.34–0.90)**							
Traditional	0.69	0.68	0.86	0.56	0.68	0.49	0.82	0.61	0.54	0.43	0.60	0.33	0.43	0.29	0.56	0.38
	(0.54–0.83)								(0.32–0.78)							
Combined	0.79	0.78	0.87	0.79	0.83	0.65	0.77	0.71	0.53	0.53	0.69	0.50	0.58	0.36	0.56	0.43
	(0.61–0.88)								(0.25–0.78)							

AUC, Area under the receiver operating characteristic; G-mean, Geometric mean score; maP, Precision of majority class; maR, Recall of majority class; maF, F-measure of majority class; miP, Precision of minority class; miR, Recall of minority class; miF, F-measure of minority class. The model with the best predictive performance was in bold.

## Discussion

In this study, a systematic analysis was firstly conducted in PET/CT radiomics, clinical, and PET/CT radiological features. Secondly, a combined model integrating the Rad-score, significant clinical and radiological features demonstrated outperformance. Thirdly, the ENN re-sampling technique improved the prediction performance of the proposed models. Our result showed that the combined model + ENN re-sampling method obtained the best performance with the AUC of 0.94 (95%CI: 0.86–0.97) and 0.75 (95%CI: 0.57–0.91) in the training and validation cohort separately.

The histologic subtype is a significant predictor in the traditional model. We grouped LUAD patients with different histologic subtype into three subgroups: the low-grade tumor, the intermediate-grade tumor, and the high-grade tumor (34). There was a significant association with increased risk of LNM in high-grade tumor (34.6% vs. 65.4%, p = 0.03). The low-grade tumor was significantly associated with no metastasis (0% *vs.* 100%, p = 0.004). Only the intermediate-grade tumor showed no statistically significant difference between LNM and non-metastasis (20% *vs.* 80%, *p* = 0.81). The histological subtyping can help to predict LNM of LUAD. But it is difficult to identify the high-risk LNM patients in the intermediate-grade tumor. We found that Rad-score is a potential metastatic predictor for the intermediate-grade LUAD, which had the significant difference in the LNM intermediate-grade tumor and non-metastatic intermediate-grade tumor (2.60 ± 1.43 *vs.* −0.72 ± 5.66, *p* <0.001) (The detail description about the relationship between LUAD histologic subtype and lymph node metastasis is shown in **Supplementary Result S4**). This revealed that Rad-score provides potential information about the tumor microenvironment which helps forecast the LNM of intermediate-grade LUAD.

The measurement of MTD and C/T ratio is affected by different nuclear medicine physicians and the reproducibility of the two characteristics is limited. This was validated in the present study that ICC of MTD and C/T ratio between different nuclear medicine physicians were 0.69 and 0.83, respectively. The morph_av and the stat_median were the optimal correlated radiomics features which replace these two radiological characteristics, presenting high robustness with the ICC values of 0.92 and 0.93 separately. The morph_av obtained significantly higher performance than MTD (AUC, 0.75 ± 0.06 *vs.* 0.65 ± 0.08, *p* <0.001), and stat_median showed significantly superior performance than C/T ratio (AUC, 0.75 ± 0.06 *vs.* 0.73 ± 0.05, *p* <0.001) (**Supplementary Tables S4, S5**). The morph_av feature is the surface to volume ratio of tumor which calculates over 3D tumor volume. The stat_median feature represents the median of voxels intensities within the tumor. MTD and C/T ratio only supplies one-dimensional information for tumor, whereas the radiomics features take into account the physical characteristics of tumor in three-dimensional that can reflect the spatial complexity for tumor. CT radiomics signature outperformed Lasso-CT features both in the train [AUC, 0.85 (95%CI: 0.74–0.91) *vs.* 0.84 (95%CI: 0.72–0.91); *p* = 0.64] and validation cohort [AUC, 0.62 (95%CI: 0.41–0.76) *vs.* 0.59 (95%CI: 0.39–0.74); *p* = 0.07]. Thus, we suggested that using well-established and benchmarked radiological factors to find the correlated features, and performing the cross-validation to determine the optimal correlated feature, is an effective approach to identify high predictive value features.

**Figure 7:**
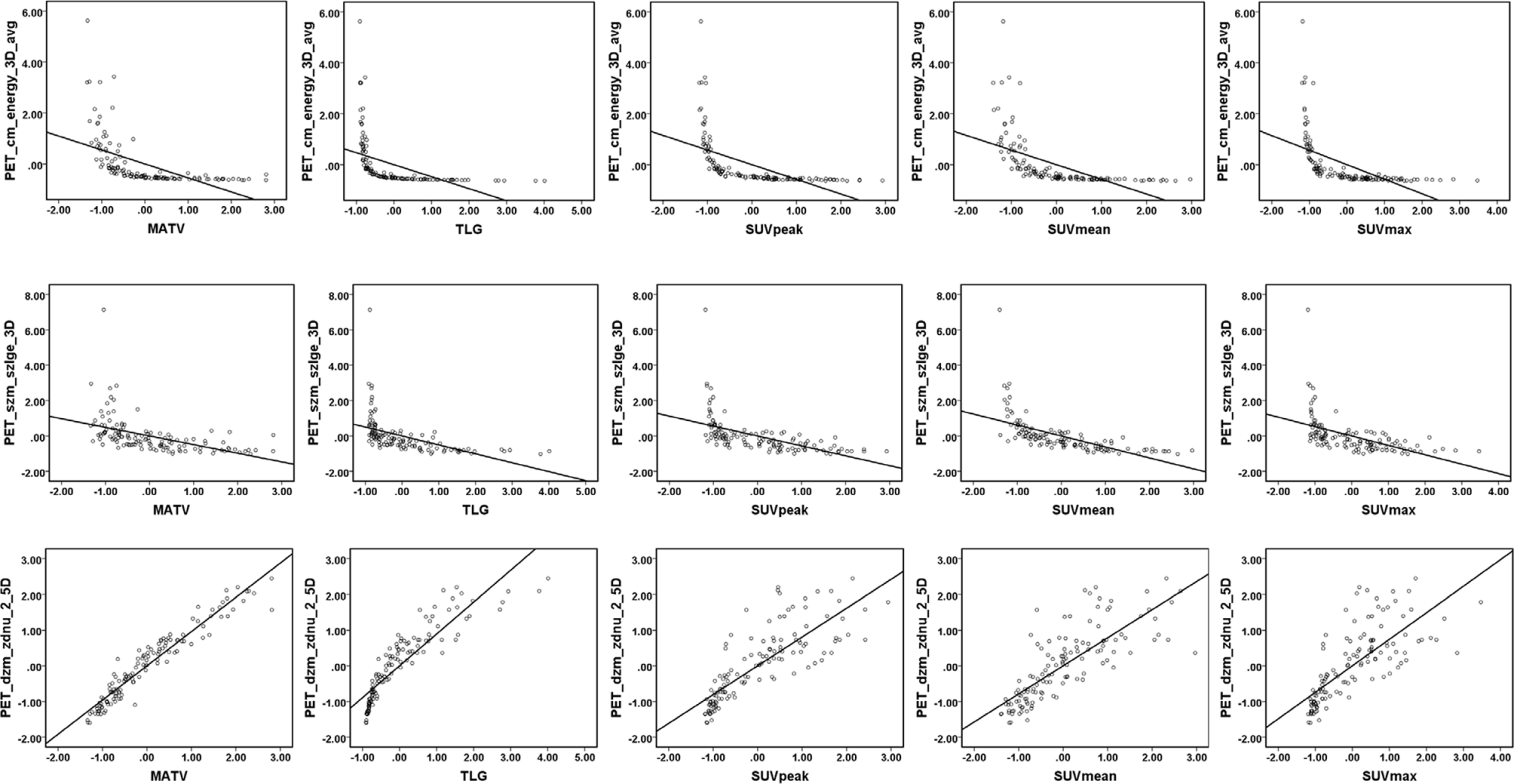
Linear regression for PET parameters and PET texture features. Linear regression and Pearson correlation were analyzed in the training cohort. A moderate negative correlation between all conventional PET parameters (MATV, TLG, SUVpeak, SUVmean, SUVmax) with PET_cm_energy_3D_avg (*r*= −0.55, −0.47, −0.58, −0.58, and −0.57, respectively) and PET_szm_szlge_3D (*r*= −0.49, −0.51, −0.56, −0.62, and −0.53, respectively). A strong positive correlation between all conventional PET parameters (MATV, TLG, SUVpeak, SUVmean, and SUVmax) with PET_dzm_zdnu_2_5D (*r*= 0.96, 0.89, 0.81, 0.79, and 0.74, respectively).

**Figure 8:**
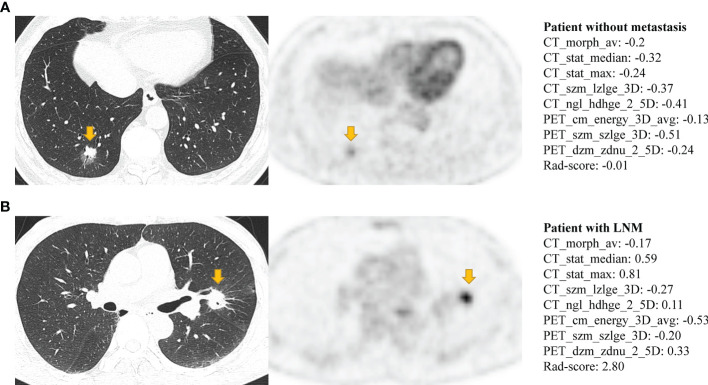
The CT and PET image of **(A)** a 50-year-old man with lung adenocarcinoma without occurring metastasis and **(B)** a 75-year-old man with lung adenocarcinoma with occurring lymph node metastasis. Both **(A, B)** have lobulation, vessel convergence, spiculatio, but difference in Rad-score. Patient **(A)** has a lower Rad-score which corresponds to show a smaller tumor, ground-glass component, slightly vague margin, less density and more homogeneous in CT image, and the slight FDG metabolism in PET image (MATV: 5.59; TLG: 8.84; SUVpeak: 2.02; SUVmean: 1.63; SUVmax: 3.01). Patient **(B)** has a higher Rad-score which corresponds to show a bigger tumor, solid component, clear margin, more density and less homogeneous in CT image, and the intense FDG metabolism in PET image (MATV: 11.58; TLG: 26.11; SUVpeak: 6.95; SUVmean: 2.27; SUVmax: 11.1).

The Rad-score consisted of the eight best-performing radiomics features, which correspond to the morphology, intensity-based statistical and texture feature. The present result indicated that CT_morph_av was a lymph node metastasis predictor of lung adenocarcinoma, as confirmed by Yang et al. (21), showing that less favorable tumors have bigger volume and clear margin. The CT_stat_median and CT_stat_max describe the median or maximum intensities within the lesion, respectively. Higher median and maximum values reflect a more solid component in the tumor. This finding was consistent with the previous study that the lung adenocarcinoma with high values of the median and maximum intensities was considered to be more aggressive (35). The CT_szm_lzlge_3D and CT_ngl_hdhge_2_5D belong to the GLSZM feature (quantify the number of linked voxels that have an identical gray level intensity) and NGLDM feature (investigate the coarseness of the overall texture) separately, the higher value of them is generally related to more heterogeneity. The CT texture features of lung cancer have been reported to associate with the markers of hypoxia and angiogenesis (36). However, the correlation between the PET texture feature and biological characteristics is still unclear. The previous study has reported that the PET texture feature plays an important role in disease diagnosis, prognosis, and treatment response prediction (37). Texture features can describe the tumor heterogeneity in PET image, which refers to the variability in the distribution of radiopharmaceutical uptake (38). Three PET texture features were selected in the present study. **Figure 7** shows a moderate negative relationship for all conventional PET parameters with PET_cm_energy_3D_avg and PET_szm_szlge_3D, and a strong positive relationship with PET_dzm_zdnu_2_5D. Thus, it can be inferred that the lower value of PET_cm_energy_3D_avg and PET_szm_szlge_3D, and the higher value of PET_dzm_zdnu_2_5D will reflect to higher glucose uptake. **Table 5** indicates that patients with LNM will have higher values of all selected CT features and PET_dzm_zdnu_2_5D and lower value of PET_cm_energy_3D_avg and PET_szm_szlge_3D. From the analysis of this work, we deduce that the tumor with bigger, more solid components, less homogeneity and more radiopharmaceutical uptake will tend to occur in lymph node metastasis. **Figure 8** shows the CT and PET image of a tumor without metastasis and another tumor with LNM.

**Table 5:** The radiomics predictor of patients with or without LNM in the training and validation cohort.

Radiomics predictor	Training cohort		Validation cohort	
	Non-metastasis	LNM	Non-metastasis	LNM
CT_morph_av	0.18 ± 1.00	−0.74 ± 0.54	0.14 ± 1.23	−0.52 ± 0.59
CT_stat_median	−0.18 ± 1.02	0.70 ± 0.50	−0.18 ± 1.00	0.15 ± 1.03
CT_stat_max	−0.18 ± 0.98	0.71 ± 0.66	−0.31 ± 1.28	0.27 ± 0.51
CT_szm_lzlge_3D	−0.16 ± 0.53	0.63 ± 1.87	−0.12 ± 0.46	−0.12 ± 0.26
CT_ngl_hdhge_2_5D	−0.22 ± 0.88	0.88 ± 0.97	0.17 ± 1.40	0.18 ± 0.80
PET_cm_energy_3D_avg	0.13 ± 1.07	−0.53 ± 0.10	0.78 ± 2.94	−0.47 ± 0.20
PET_szm_szlge_3D	0.14 ± 1.06	−0.57 ± 0.30	0.43 ± 2.81	−0.27 ± 0.51
PET_dzm_zdnu_2_5D	−0.22 ± 0.92	0.89 ± 0.80	0.07 ± 1.35	0.20 ± 0.87
Rad-score	−0.79 ± 4.65	3.18 ± 1.20	−2.99 ± 10.93	1.77 ± 1.46

The average value and the standard deviation of radiomics predictors were reported. LNM, Lymph node metastasis.

Re-sampling methods include three classes: over-sampling, under-sampling, and hybrid methods. Over-sampling strategy creates examples of the minority class to balance the dataset. It helps to improve the performance of models, but presents the drawback of overfitting and introduces additional noise (39). Under-sampling strategy addresses the imbalance problem by eliminating the members of the majority class, showing an advantage in saving computation time, while will discard potentially useful information (39). Hybrid method combines the over-sampling of the minority class with under-sampling of the majority class (39). Previous studies have reported the successful application of various re-sampling methods in radiomics analysis (31, 40). Xie et al. (31) examined the effect of ten re-sampling techniques based radiomics model on the prognostication performance of head and neck cancer; the result demonstrated that the ADASYN re-sampling method performed best for overall survival prediction, while bSMOTE is the optimal re-sampling technique for disease-free survival. Park et al. (40) applied the ROS and SMOTE re-sampling techniques based on radiomics analysis to predict the grade and histological subtype of meningiomas, where the best performance was yielded by using the SMOTE re-sampling. The selection of the best re-sampling technique is complicated. Since the effectiveness of re-sampling techniques depends on intrinsic properties of the dataset, such as dataset size and dimensionality, imbalance ratio, overlapping between classes or borderline samples (41). In the present study, the majority class and minority class have close properties, such as both of them are clinical T1 stage lung adenocarcinoma. That may explain why the over-sampling strategy did not obtain better results than the ENN re-sampling techniques. The result of the Monte Carlo cross-validation demonstrated that performing feature selection before data re-sampling could achieve advanced predictive performance than the reversed sequence (AUC 0.76 ± 0.06 *vs.* 0.70 ± 0.07, *p* <0.001) (**Supplementary Table S6**). It may be because feature selection applying raw data can purely reflect the relationship between features and clinical problems. The maF and miF represent the prediction ability of the models for the majority and minority class separately. Except for the maF (**Supplementary Figure S2**), the miF (**Supplementary Figure S3**) showed a consistent trend with G-mean (**Figure 6B**). The improvement of G-mean was more dependent on the accurate prediction of the minority class. The re-sampling techniques veritably promoted the predictive performance of the models for the minority class.

The solid component of LUAD is more invasive, where patients with a pure solid tumor often have poor outcome (42). Our study also confirmed that the efficient performance of the radiomics model and traditional model was primarily relying on the accurate LNM prediction in the non-solid tumor (**Supplementary Result S5**). The LNM of solid tumors was more difficult to predict. Encouragingly, the radiomics model displayed an optimum predictive performance in the solid tumor subgroup, which was a potential tool to distinguish more aggressive tumors form solid LUAD.

Although it is widely accepted that systematic lymph node dissection for patients with early-stage lung cancer is important, controversy still exists (7, 8). Extensive lymph node dissection can provide accurate lymph node staging while causing an increased risk of complications and prolonging operation time, which is particularly detrimental to elderly patients with cardiopulmonary dysfunction (43). This controversy is even greater among the patients with clinical-stage T1 lung adenocarcinoma who are diagnosed with clinical lymph node-negative and presented as less aggressive tumors, such as pure ground-glass opacity or mixed ground-glass opacity, which have a very low probability of lymph node metastasis (44). If there is a non-invasive examination that can accurately predict lymph node metastasis before operation with a highly accurate, it will be very important for treatment guidance. Our model (ENN + Combined) exhibited a favorable ability to discriminate the patients with LNM with AUCs of 0.94 and 0.75, true positive rate of 0.88 and 0.78, in the training and validation cohorts, respectively. Thus, the model could provide LNM risk stratification, which would assist the clinicians to make the treatment decision. For example, patients with a high risk of LNM will be recommended for accepting more radical and complete nodal dissection and careful surveillance and patients with low risk of LNM were suitable for lymph node sampling. As a promising adjuvant tool, it could guide therapeutic strategies and personalize decision-making.

This work had several limitations. First, in the 35 LNM cases, 32 patients with LNM were confirmed by pathological examination, while 3 patients with LNM were identified by PET/CT and confirmed by multiple time points of enhanced contrast CT during following-up. It would increase the probability of false positive. Second, the number of patients with lymph node metastasis and non-metastasis was very imbalanced. Although we performed re-sampling technique to adjust the data distribution in training group, a large sample size of LNM cases is still needed for future studies. Third, our work is a retrospective study on single-institution, and an external validation is necessary to confirm our findings and assess the generalizability. Finally, manual segmentation was applied in our study and automatic segmentation method should be developed in further analysis.

In conclusion, the combined model connected with ENN re-sampling method had a powerful ability to predictive the lymph node metastasis of LUAD with stage T1. The approach that we reported above might assist the clinician to make the individualized therapy strategies.

## Data Availability Statement

Publicly available datasets were analyzed in this study. This data can be found here: https://github.com/JieqinLv/Predictive-metastasic-LUAD.

## Ethics Statement

This retrospective study was approved by the Institutional Review Boards, Nanfang Hospital, Southern Medical University, and the need for informed consent for patients were waived. Written informed consent was not obtained from the individual(s) for the publication of any potentially identifiable images or data included in this article.

## Author Contributions

All authors listed have made a substantial, direct and intellectual contribution to the work, and approved it for publication.

## Funding

This work was supported by the National Natural Science Foundation of China under grants 81871437, 12026601, the Guangdong Province Universities and Colleges Pearl River Scholar Funded Scheme (LL, 2018), the Guangdong Basic and Applied Basic Research Foundation under grants 2019A1515011104, 2020A1515110683, and 2021A1515011676, and the China Postdoctoral Science Foundation funded project 2020M682792.

## Conflict of Interest

The authors declare that the research was conducted in the absence of any commercial or financial relationships that could be construed as a potential conflict of interest.

## Publisher’s Note

All claims expressed in this article are solely those of the authors and do not necessarily represent those of their affiliated organizations, or those of the publisher, the editors and the reviewers. Any product that may be evaluated in this article, or claim that may be made by its manufacturer, is not guaranteed or endorsed by the publisher.
