# Supplementary material for: Imbalanced Data Correction Based PET/CT Radiomics Model for Predicting Lymph Node Metastasis in Clinical Stage T1 Lung Adenocarcinoma
Source: Front Oncol. 2022 Jan 28;12:788968. doi: 10.3389/fonc.2022.788968 (PMC8831550; doi:10.3389/fonc.2022.788968)
Supplement: Supplementary file 1 [file DataSheet_1.docx]

***Supplementary Material***

| **1.** | **Supplementary Methods** | | **Pages 3-8** |
| --- | --- | --- | --- |
| Method S1 | | Lymph node metastasis evaluation and histopathologic classification criterion | Page 3 |
| Method S2 | | ^18^F-FDG PET/CT image acquisition | Page 3 |
| Method S3 | | Tumor segmentation | Page 3, 4 |
| Method S4 | | MTD and C/T ratio measurement | Pages 4 |
| Method S5 | | Feature extraction | Page 4 |
| Method S6 | | Inter-observer reproducibility analysis | Pages 4 |
| Method S7 | | Re-sampling methods | Pages 5 |
| Method S8 | | Radiomics features selection | Pages 6, 7 |
| Method S9 | | Evaluation metric formulas | Pages 7 |
| Figure SM1 | | Tumor segmentations on CT image and PET image | Pages 7 |
| Figure SM2 | | The process of determining the optimal feature correlated with MTD and the optimal feature correlated with C/T ratio | Pages 7, 8 |
| Table SM1 | | Parameter configurations for PET/CT image processing and texture computation | Pages 8 |
| **2. Supplementary Results** | | | **Pages 9-18** |
| Result S1 | | Radiomics feature selection result | Pages 9 |
| Result S2 | | Formula of Rad-score | Pages 9, 10 |
| Result S3 | | The performance of each model combined with re-sampling techniques | Pages 10 |
| Result S4 | | The relationship between LUAD histologic subtype and lymph node metastasis | Pages 10, 11 |
| Result S5 | | Confusion matrixes of the radiomics model and traditional model | Pages 11 |
| Table SR1 | | Number of PET features remained after each step in Part 1 | Pages 11 |
| Table SR2 | | Number of CT features remained after each step in Part 1 | Pages 11, 12 |
| Table SR3 | | Number of PET features remained after each step in Part 2 | Pages 12 |
| Table SR4 | | Number of CT features remained after each step in Part 3 | Pages 12 |
| Table SR5 | | The Morphological features that correlated with MTD | Pages 12, 13 |
| Table SR6 | | The Intensity-based statistical features that correlated with C/T ratio | Pages 13 |
| Table SR7 | | Pearson correlation analysis between LASSO-CT features and morph_av feature or stat_median feature | Pages 13 |
| Table SR8 | | The predictive performance of the selected single feature | Pages 13 |
| Table SR9 | | The predictive performance of radiomics model combined with re-sampling techniques | Pages 13, 14 |
| Table SR10 | | The predictive performance of traditional model combined with re-sampling techniques | Pages 14, 15 |
| Table SR11 | | The predictive performance of combined model combined with re-sampling techniques | Pages 15 |
| Table SR12 | | The number of patients with or without metastasis in the histologic subtype subgroup in the overall, training and validation cohort | Pages 15, 16 |
| Figure SR1 | | The distribution of Rad-score of the intermediate-grade tumor with or without metastasis | Pages 16 |
| Figure SR2 | | Confusion matrixes of the radiomics model and traditional model | Pages 17, 18 |
| **3.** | **Supplementary Figures** | | **Pages 18-19** |
| Figure S1 | | The receiver operating characteristic (ROC) curves of the proposed models in the solid tumor subgroup | Page 18, 19 |
| Figure S2 | | The F-measure of the majority class (maF) of the proposed models with and without re-sampling techniques | Pages 19 |
| Figure S3 | | The F-measure of the minority class (miF) of the proposed models with and without re-sampling techniques | Page 19 |
| **4.** | **Supplementary Tables** | | **Pages 19-22** |
| Table S1 | | Data distribution of the training cohort in different re-sampling methods | Page 20 |
| Table S2 | | The results of univariate logistic regression analysis of the clinicopathological and radiological features | Page 20 |
| Table S3 | | Patient characteristics in the solid tumor subgroup | Page 20, 21 |
| Table S4 | | Comparing the predictive ability between MTD and morph_av radiomics feature | Page 22 |
| Table S5 | | Comparing the predictive ability between C/T ratio and stat_median radiomics feature | Pages 22 |
| Tables S6. | | Comparing the predictive performance of different order between feature selection and re-sampling data in the machine learning pipeline in radiomics analysis | Pages 22 |
| **5.** | **References** | | **Pages 23-24** |

**1. Supplementary Method**

**Method S1. Lymph node metastasis evaluation and histopathologic classification criterion**

A total of 183 patients were enrolled, including 148 non-metastasis cases and 35 lymph node metastasis cases. In the 35 LNM cases, 32 patients with LNM were confirmed by pathological examination, while 3 patients with LNM were identified by PET/CT and also confirmed by multiple time points of enhanced contrast CT during following-up. Except for 10 non-metastasis cases, 173 cases (include 133 non-metastasis and 35 lymph node metastasis cases) had undergone lymph node dissection. Histopathologic classification criterion of lung adenocarcinoma was proposed by the International Association for the Study of Lung Cancer, the American Thoracic Society and the European Respiratory Society (IASLC/ATS/ERS) (1). The overall stage is based on the American Joint Committee on Cancer (AJCC) eighth edition cancer staging manual.

**Method S2. ^18^F-FDG PET/CT image acquisition**

Whole body ^18^F-FDG PET/CT scanning was performed on a Biograph mCT scanner (Siemens, Germany) following the European Association of Nuclear Medicine (EANM) procedure guidelines (2). All patients need to fast for more than 6 h before the examination. The blood glucose level was maintained below 11.1 mmol/L before an intravenous injection with 271~410 MBq of ^18^F-FDG (produced by TRACERlabFX2N, purity > 95%; GE Healthcare) depending on the patient’s weight (~ 5.55MBq/kg), then the scanning was initiate after 60-min uptake (56±7 min, range 51-69 min).

First, CT scan was performed with bulb voltage of 120 kV and automatic tube current modulation (64-321 mA, pitch 0.8 mm), the CT images were reconstructed using standard lung reconstruction algorithm (parameters: in-plane resolution 0.82 × 0.82 mm2, slice thickness 1.5 mm, slice spacing 1 mm, convolution kernel B70f). Next, the PET scan was acquired in a three-dimensional mode with a speed of 2 min/ bed. The ordered-subset expectation maximization (OSEM) algorithm with point spread function (PSF) modeling (3 iterations, 21 subsets) was used to reconstruct the time-of-flight (TOF) -PET data and PET images were corrected for attenuation by using the transmission CT data. Post reconstruction smoothing filtering via a 4-mm full-width-at-half-maximum (FWHM) Gaussian filter to spatially smooth intensities. All PET imaging data (in-plane resolution 4.07 × 4.07 mm2; slice thickness 5 mm; slice spacing 3 mm) were converted into standardized uptake values (SUVs) based on the patient body weight.

**Method S3. Tumor segmentation**

ITK-SNAP software (version 3.6.0; [www.itksnap.org](http://www.itksnap.org)) was utilized for manual three-dimensional segmentation. The primary tumor on the PET and CT images were segmented independently. Supplementary Methods Figure SM1 shows the tumor segmentation.

**Method S4. MTD and C/T ratio measurement**

Maximum tumor diameter (MTD) and consolidation-to-tumor (C/T) ratio were measured manually on CT images with lung window (window width 1200HU; window level − 600HU) using the ruler tool on the Syngo MMWP workstation (Siemens, Germany), which allows the operator to draw lines on regions of interest (ROI). The tumor diameters were measured in the transverse, coronal, and sagittal planes, and the largest diameter in the three planes was defined as MTD. The area with increased opacification that completely obscured underlying vascular markings was considered a consolidation component. The C/T ratio was defined as the maximum consolidation (C) diameter divided by the maximum tumor (T) diameter in the transverse plane.

**Method S5.** **Feature extraction**

Radiomics features were calculated on both CT and PET images within the whole tumor region. Feature extraction was conducted using the standardized environment for radiomics analysis (SERA) software (3,4) based on MATLAB platform, which can ensure the reproducibility of the extracted radiomics feature and match the benchmarks of image biomarker standardization initiative (IBSI) guidelines (5,6). A total of 487 radiomics features were extracted and can be grouped as 11 different types: (1) 29 morphological features; (2) 2 local intensity features; (3) 18 intensity-based statistical features; (4) 23 intensity histogram features; (5) 7 intensity-volume histogram features; and 408 texture features including (6) 150 Gray level co-occurrence matrix (GLCM) features, (7) 96 Gray level run length matrix (GLRLM) features, (8) 48 Gray level size zone matrix (GLSZM) features, (9) 48 Gray level distance zone matrix (GLDZM) features, (10) 15 neighborhood gray tone difference matrix (NGTDM) features, and (11) 51 neighborhood gray level dependence matrix (NGLDM) features. The details of image pre-processing for feature extraction and feature definitions are explained in the IBSI document (6). Parameter configurations for PET/CT image processing and texture computation are described in Supplementary Methods Table SM1. The PET and CT images were resampled to isotropic 3×3×3 mm3 and 1×1×1 mm3 voxel size separately using trilinear interpolation algorithm, and grey levels were discretized by a fixed bin width of 0.1 SUV and 25 HU separately (7).

**Method S6.** **Inter-observer reproducibility analysis**

MTD, C/T ratio, and tumor segmentation were operated by an expert nuclear medicine physician with 4 years of work experience. Each result was reviewed by a senior with 10 years of work experience. To evaluate inter-observer reproducibility, another measurement and segmentation were performed on 50 randomly selected patients from the training cohort by a nuclear medicine physician with 3 years of work experience.

**Method S7.** **Re-sampling methods**

***1. Over-sampling:***

I. Random over-sampling (ROS): the mostly naive approach is to create new samples by randomly sampling the current samples.

II. Adaptive Synthetic (ADASYN) (8): the idea of ADASYN is to use a weighted distribution for different examples of the minority class depending on their difficulty in learning. It produces new data for minority class examples that are difficult to learn, making the classification decision boundary toward the difficult examples.

III. Synthetic Minority Oversampling Technique (SMOTE) (9): each sample of minority class was oversampled and generating synthetic samples based on the k nearest neighbors of each minority class samples. The number of k nearest neighbors can randomly selected. This algorithm can make the decision region of the minority class more generalizable.

IV. Borderline SMOTE (bSMOTE) (10): the samples on or nearby the borderline of each class are easy to misclassified (those samples called borderline samples). The technique base on SMOTE, only over-sample or strengthen the borderline minority samples.

***2. Under-sampling:***

I. Random under-sampling (RUS): this technique randomly deletes samples for the majority class.

II. NearMiss (NM) (11): the essential idea is to use heuristic rules of the nearest neighbor approach to compute the mean distance for sample selection in the majority class.

III. Tomek link (TL) (12): the concept of Tomek link is described as follow: existing an instance pair (x_i,x_j), where x_i∈S_minority, x_j∈S_majority and d(x_i,x_j ) is the distance between x_i and x_j, if there is not instance x_k such that d(x_i,x_k )<d(x_i,x_j ) or d(x_j,x_k )<d(x_i,x_j ), then the instance pair (x_i,x_j) is named Tomek link. Two samples from a Tomek link means either one of them is noise or both are near the borderline. This technique removes overlapping samples of the majority class.

IV. Edited Nearest Neighbours (ENN) (13): this technique based on Nearest neighbor rules and remove examples which do not agree with the majority of its k nearest neighbors (with k"=3", generally). It produces a smoother decision borderline by editing out noisy and close-boundary samples.

***3. Hybrid method:***

SMOTE technique creates noisy examples that spread on both minority and majority class. To solve the overgeneralization problem of SMOTE the hybrid methods were mentioned. The hybrid method integrates under-sampling and over-sampling by increasing samples of minority class and decreasing samples of majority.

I. SMOTE-TL (14): this technique applies SMOTE to produce synthetic data of minority class and deletes the instances that compose of Tomek link.

II. SMOTE-ENN (15): the idea of SMOTE-ENN is similar to SMOTE-TL. This approach uses SMOTE to create new data, discard noisy data by ENN.

The parameters setting in the present study:

| Re-sampling techniques | Parameters |
| --- | --- |
| ADASYN | n_neighbors=5 (The number of nearest neighbors that used to construct synthetic samples) |
| SMOTE | n_neighbors=5 (The number of nearest neighbors that used to construct synthetic samples) |
| bSMOTE | n_neighbors=5 (The number of nearest neighbors that used to construct synthetic samples);  m_neighbors=10 (The number of nearest neighbors that use to determine if a minority sample is in danger);  kind=borderline-1 (The type of SMOTE algorithm) |
| NM | n_neighbors=3 (The size of the neighborhood that used to compute the average distance to the minority point samples) |
| ENN | n_neighbors=3 (The size of the neighborhood that used to compute the average distance to the minority point samples);  kind_sel=all (The strategy to exclude sample) |
| SMOTE-TL | n_neighbors=5 (The number of nearest neighbors that used to construct synthetic samples) |
| SMOTE-ENN | n_neighbors=5 (The number of nearest neighbors that used to construct synthetic samples); n_neighbors=3 (The size of the neighborhood that used to compute the average distance to the minority point samples); kind_sel=all (The strategy to exclude sample) |

**Method S8.** **Radiomics features selection**

All of the radiomics features were standardized by $Z$score transformation. In our study, the process of feature selection consisted of three parts.

Part 1, PET and CT radiomics features were initially selected according to the following three steps. Step 1, features with an ICC higher than 0.75 were identified as robust features and retained; step 2, features with median absolute deviations (MAD) < 0.05 were considered to be non-informative and discarded; step 3, features with $p$<0.01 (considering the false discovery rate correction) in univariate logistic regression analysis were considered to be associated with LNM metastasis, and were selected as the remaining PET or CT radiomics features for further analysis.

Part 2, PET radiomics features selection. Pearson correlation coefficient was first calculated among all features, and for each feature pair with an absolute correlation coefficient higher than 0.8, the feature that obtained higher $p$ value in univariate analysis was removed. The regularized multivariate logistic regression with the least absolute shrinkage and selection operator (LASSO) penalty was used to obtain the optimal feature combination (16). Tenfold cross-validation was repeated 50 times to minimize the selection bias of the regularization parameter. Features that appeared most frequently were used to generate PET radiomics signature.

Part 3, CT radiomics feature selection. MTD and C/T ratio were prognostic factors for lung adenocarcinoma, however, due to their high inter-observer variability, this study tries to find two alternative features that extracted automatically and with high robustness. First, Pearson correlation coefficient was calculated between MTD and CT morphological features, C/T ratio and CT intensity-based statistical features, respectively. Radiomics features with Pearson correlation coefficient > 0.75 were selected as candidate morphological features and candidate intensity-based statistical features. Univariate logistic regression analysis was performed for each candidate morphological feature and candidate intensity-based statistical feature, using 50 times 5-fold cross-validation in the training data, and the predictive performance was assessed using area under the curve (AUC). Two features correlated with MTD and C/T ratio separately that achieving the maximum mean validation AUC in the 250 validation rounds were selected from the candidate features. Secondly, we need to select beneficial features from the remaining CT features in Part 1. The feature selection method was the same as PET radiomics feature selection method (described in Part 2), removing the redundancy features, then determining the effective feature combination (we called “LASSO-CT features” in this study). Thirdly, the LASSO-CT features and two optimal features correlated with MTD and C/T individually may have potential correlation information. We applied Pearson correlation analysis in which a threshold higher than 0.8 to remove the redundancy LASSO-CT features. The optimal correlated feature with MTD, the optimal correlated feature with C/T ratio, and the non-redundancy LASSO-CT features were consolidated into the CT radiomics signature.

In the end, PET/CT radiomics signature was developed by combing two signatures of CT and PET images. The PET/CT radiomics model was developed by the multivariable logistic regression. Each patient’s Rad-score was calculated using a linear combination of selected radiomics feature weighted by their respective coefficients. Supplementary Methods Figure SM2 illustrated the process of determining the two optimal features that correlated with MTD and C/T ratio individually.

**Method S9.** **Evaluation metric formulas**

| **Actual Class** | **Predicted Class** | |
| --- | --- | --- |
|  | Non-metastasis = Majority | LNM = Minority |
| Non-metastasis = Majority | TN | FP |
| Metastasis = Minority | FN | TP |

$$\boldsymbol{Geometric} \boldsymbol{mean} \boldsymbol{score}\mathbf{(}\boldsymbol{G}\mathbf{-}\boldsymbol{mean}\mathbf{)=}\sqrt{\frac{\boldsymbol{TP}}{\boldsymbol{TP}\mathbf{+}\boldsymbol{FN}}\boldsymbol{\cdot}\frac{\boldsymbol{TN}}{\boldsymbol{TN}\mathbf{+}\boldsymbol{FP}}}$$

$\boldsymbol{Precision}_{\boldsymbol{Minority}} \left( \boldsymbol{P}_{\boldsymbol{mi}} \right)\mathbf{=}\frac{\boldsymbol{TP}}{\boldsymbol{TP}\mathbf{+}\boldsymbol{FP}}$ $\boldsymbol{Precision}_{\boldsymbol{Majority}} \left( \boldsymbol{P}_{\boldsymbol{ma}} \right)\mathbf{=}\frac{\boldsymbol{TN}}{\boldsymbol{TN}\mathbf{+}\boldsymbol{FN}}$

$\boldsymbol{Recall}_{\boldsymbol{Minority}} \left( \boldsymbol{R}_{\boldsymbol{mi}} \right)\mathbf{=}\frac{\boldsymbol{TP}}{\boldsymbol{TP}\mathbf{+}\boldsymbol{FN}}$ $\boldsymbol{Recall}_{\boldsymbol{Majority}} \left( \boldsymbol{R}_{\boldsymbol{ma}} \right)\mathbf{=}\frac{\boldsymbol{TN}}{\boldsymbol{TN}\mathbf{+}\boldsymbol{FP}}$

$${\boldsymbol{F}\mathbf{-}\boldsymbol{measure}}_{\boldsymbol{Minority}} \left( \boldsymbol{F}_{\boldsymbol{mi}} \right)\mathbf{=}\frac{\mathbf{2}\boldsymbol{P}_{\boldsymbol{mi}}\boldsymbol{R}_{\boldsymbol{mi}}}{{\boldsymbol{P}_{\boldsymbol{mi}}\mathbf{+}\boldsymbol{R}}_{\boldsymbol{mi}}}\mathbf{=}\frac{\mathbf{2}\boldsymbol{TP}}{\mathbf{2}\boldsymbol{TP}\mathbf{+}\boldsymbol{FN}\mathbf{+}\boldsymbol{FP}}$$

$${\boldsymbol{F}\mathbf{-}\boldsymbol{measure}}_{\boldsymbol{Majority}} \left( \boldsymbol{F}_{\boldsymbol{ma}} \right)\mathbf{=}\frac{\mathbf{2}\boldsymbol{P}_{\boldsymbol{ma}}\boldsymbol{R}_{\boldsymbol{ma}}}{{\boldsymbol{P}_{\boldsymbol{ma}}\mathbf{+}\boldsymbol{R}}_{\boldsymbol{ma}}}\mathbf{=}\frac{\mathbf{2}\boldsymbol{TN}}{\mathbf{2}\boldsymbol{TN}\mathbf{+}\boldsymbol{FN}\mathbf{+}\boldsymbol{FP}}$$

**Figure SM1.** **Tumor segmentations on CT image and PET image**


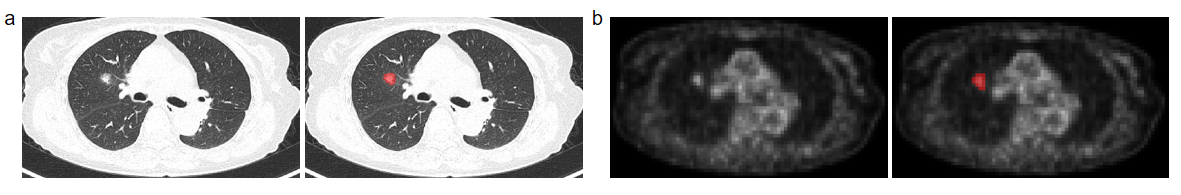
**Figure SM1.** Tumor segmentations (in red) on (a) CT image and (b) PET image

**Figure SM2.** **The process of determining the optimal feature correlated with MTD and the optimal feature correlated with C/T ratio**

**
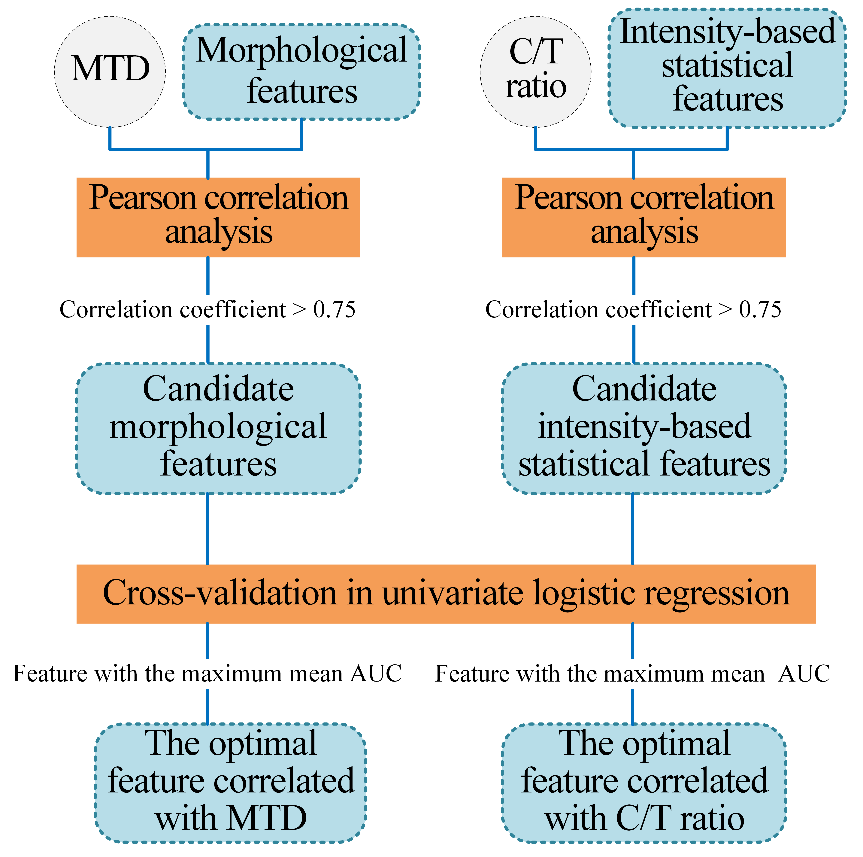
**

**Figure SM2.** The process of determining the optimal feature correlated with MTD and the optimal feature correlated with C/T ratio

**Table SM1. Parameter configurations for PET/CT image processing and texture computation**

**Table SM1.** Parameter configurations for PET/CT image processing and texture computation

| Parameter | PET | CT |
| --- | --- | --- |
| Approach | 2D, 2.5D, 3D | 2D, 2.5D, 3D |
| Interpolation |  |  |
| Resampled voxel spacing (mm) | 3×3×3 | 1×1×1 |
| Interpolation method | trilinear | trilinear |
| Intensity rounding | nearest integer | nearest integer |
| ROI interpolation method | trilinear | trilinear |
| ROI partial mask volume | 0.5 | 0.5 |
| Re-segmentation |  |  |
| Range (SUV or HU) | [0, inf] | [-1000, 400] |
| Outlier filtering | no | no |
| Discretisation |  |  |
| Texture and IH | FBS: 0.1 SUV | FBS: 25 HU |
| IVH | FBS: 0.1 SUV | FBS: 2.5 HU |
| Texture parameters |  |  |
| GLCM, NGTDM, NGLDM distance | 1 | 1 |
| GLSZM, GLDZM linkage distance | 1 | 1 |
| NGLDM coarseness | 0 | 0 |

*IH*, intensity histogram features; *FBS*, fixed bin size; *IVH*, intensity-volume histogram features; *GLCM*, gray-level co-occurrence matrix; *NGTDM*, neighborhood gray tone difference matrix; *NGLDM*, neighborhood grey level dependence matrix; *GLSZM*, gray level size zone matrix; *GLDZM*, gray level distance zone matrix.

**2. Supplementary Result**

**Result S1. Radiomics feature selection result**

Part 1, after the inter-observer reproducibility analysis, there were 447 and 384 robust features were selected from PET and CT radiomics features separately. Next, 438 PET and 377 CT features were considered as having informative in the information evaluation. Last, 368 PET and 272 CT features were found to be significantly related to metastasis with the univariate analysis and were regarded as the PET and CT remaining features separately. Supplementary Result Table SR1 described the number of PET features that remained after each step in Part 1. Supplementary Result Table SR2 described the number of CT features that remained after each step in Part 1.

Part 2, 368 remaining PET features were adopted for the following analysis. After redundancy reduction, 5 PET features were selected. Ultimately, the effective feature combination that including 3 PET features were determined with the LASSO. Supplementary Result Table SR3 showed the number of PET features that remained after each step in Part 2.

Part 3, the number of 272 remaining CT features, 29 morphological features and 18 intensity-based statistical features were investigated in this part. Supplementary Result Table SR4 described the number of CT features that remained after each step in Part 3. 7 morphological features and 5 intensity-based statistical features were significantly associated with MTD and C/T ratio respectively (Supplementary Result Table SR5, SR6). It can be inferred from the result of 50 times 5-fold cross-validation that the morph_av (Surface to volume ratio, calculated over 3D ROI volume) and stat_median (Intensity-based statistical median feature, calculated over 3D ROI volume) feature were the optimal correlated features with MTD and C/T ratio separately (Supplementary Result Table SR5, SR6). 272 remaining CT features were conducted to remove the redundancy features, then obtained the useful feature combination via LASSO (We called the “LASSO-CT feature”). 5 LASSO-CT feature have performed the Pearson correlation analysis with morph_av feature and stat_median feature separately, we remove 2 high correlated features (szm_hgze_2D and dzm_ldhge_2_5D feature) from the LASSO-CT feature (Supplementary Result Table SR7). Eventually, we obtained 5 CT radiomics feature.

In the end, 3 PET and 5 CT features were combined to the PET/CT radiomics signature. The predictive performance of the selected single feature is showed in Supplementary Result Table SR8.

**Result S2. Formula of Rad-score**

Rad-score= 0.4110*CT_morph_av + 0.1456 *CT_ stat_median + 1.1069*CT_stat_max + 0.4560*CT_szm_lzlge_3D + 0.4017*CT_ngl_hdhge_2_5D - 3.2949*PET_cm_energy_3D_avg -0.2705*PET_szm_szlge_3D + 0.0334*PET_dzm_zdnu_2_5D

**Result S3.** **The performance of each model combine with re-sampling techniques**

Supplementary Result Tables SR9 shows the predictive performance of radiomics signature combined with 10 re-sampling techniques separately. Supplementary Result Tables SR10 depict the predictive performance of traditional model combined with re-sampling techniques. The predictive performance of combined model trained with different re-sampling techniques are illustrate in Supplementary Result Tables SR11.We also compare the predictive performance of effective re-resampling technique with no-resampling in each model in the validation cohort. The ENN, RUS, SMOTE-ENN, NM and bSMOTE helped to improve the performance of radiomics model (AUC of 0.71, 0.69, 0.68, 0.68, 0.68$vs$. 0.67; $p$ =0.18, 0.58, 0.88, 0.89, 0.62). The traditional model showed better performance in ENN and ADASYN (AUC of 0.68, 0.68 $vs$. 0.67; $p$ =0.59, 0.33) than no-resampling. The combined model achieved excellent performance in ENN, RUS, bSMOTE and ROS (AUC of 0.75, 0.74, 0.72, 0.71$vs$. 0.70; $p$ =0.06, 0.11, 0.72,0.61).

**Result S4.** **The relationship between LUAD histologic subtype and lymph node metastasis**

Number of 7 histologic subtype of lung adenocarcinoma enter in the present study which include AIS, MIA, LPA, APA, PPA, SPA, and IMA. We grouped LUAD patients with different histologic subtype into three subgroups: the low-grade tumor, the intermediate-grade tumor and the high-grade tumor. The low-grade tumor consisted of AIS and MIA, the intermediate-grade comprised of LPA, APA and PPA, while SPA and IMA were considered as the high-grade tumor. The higher grade of the group indicated a poor prognosis.

In our overall patients (N=183), 24 patients in the low-grade tumor, and none of them occurred metastasis. 133 LUAD patients were in the intermediate-grade tumor group, including 26 LNM instances and 107 non-metastasis instances. 26 LUAD patients with high-grade tumor, including 9 LNM instances and 17 non-metastasis instances. The present study found no statistically significant difference between LNM and non-metastasis in the intermediate-grade tumor (20% vs. 80%, p =0.81). There was a significant association with increased risk of LNM in high-grade tumor (34.6% vs. 65.4%, p =0.03). The low-grade tumor was significantly associated with no metastasis (0% vs. 100%, p =0.004).

In the training cohort (N=130), there were 15 patients (0/15 LNM/non-metastasis) in the low-grade tumor group, 97 patients (19/78 LNM/non-metastasis) in the intermediate-grade tumor group, 18 patients (7/11 LNM/non-metastasis) in the high-grade tumor group. Similarly, only the intermediate-grade tumor showed no statistically significant difference between LNM and non-metastasis (19.6% vs. 80.4%, p =0.84). The number of patients in the validation cohort is small (N=53), no relationship was found between the histologic subtype and metastasis. Supplementary Result Table SR12 showed the number of patients with or without metastasis in the histologic subtype subgroup in the overall, training and validation cohort.

Above result illustrate that it difficult for intermediate-grade tumor to differentiate the patients with LNM. We supposed that the Rad-score is an effective metastasis predictor of intermediate-grade lung adenocarcinoma. To tested this assumption, we compare the Rad-score of patients with or without metastasis in the intermediate-grade tumor group. Supplementary Result Figure SR1 showed the distribution of Rad-score of the intermediate-grade tumor with or without metastasis. The Rad-score of intermediate-grade tumor with LNM and non-metastasis was significant difference in the whole patients (2.60±1.43 $vs.$ -0.72±5.66, $p$ < 0.001). As well as in the training cohort, the significant difference of the Rad-score was found in intermediate-grade tumor with or without metastasis (2.98±1.21 $vs.$ -0.29±4.12,$p$ < 0.001).

**Result S5. Confusion matrixes of the radiomics model and traditional model**

Supplementary Result Figure SR2 show the confusion matrixes of the radiomics model and traditional model, which depict the number of correct or incorrect lymph node metastatic prediction in the non-solid tumor and solid tumor both in the training and validation cohorts. Both the radiomics model and traditional model showed better performance on lymph node metastatic prediction for the non-solid tumor with ACCs of 0.95 and 0.94 in the training cohort, and with ACCs of 0.83 and 0.93 in the validation cohort. It was also found that the worse performance of lymph node metastatic prediction for the solid tumor by radiomics signature and traditional model, with ACCs of 0.62 and 0.66 in the training cohort, and with ACCs of 0.38 and 0.46 in the validation cohort. The results revealed that the efficient performance of the radiomics model and traditional model was primarily due to the better accurate metastatic prediction in the non-solid tumor.

**Table SR1. Number of PET features remained after each step in Part 1**

**Table SR1.** Number of PET features remained after each step in Part 1

| Feature selection steps | Mor | LI | IS | IH | IVH | GLCM | GLRLM | GLSZM | GLDZM | NGTDM | NGLDM |
| --- | --- | --- | --- | --- | --- | --- | --- | --- | --- | --- | --- |
| Before selection | 29 | 2 | 18 | 23 | 7 | 150 | 96 | 48 | 48 | 15 | 51 |
| Inter-observer reproducibility | 14 | 2 | 17 | 17 | 7 | 142 | 95 | 47 | 47 | 13 | 46 |
| Information evaluation | 14 | 2 | 17 | 17 | 7 | 136 | 95 | 44 | 47 | 13 | 46 |
| Univariate analysis | 12 | 2 | 15 | 15 | 4 | 111 | 91 | 40 | 45 | 9 | 42 |

*Mor*, morphological features; *LI*, local intensity (peak) features; *IS,* intensity-based statistical features; *IH*, intensity histogram features; *IVH*, intensity-volume histogram features; *GLCM*, gray-level co-occurrence matrix; *GLRLM*, gray-level run-length matrix; *GLSZM*, gray level size zone matrix; *GLDZM*, gray level distance zone matrix; *NGTDM*, neighborhood gray tone difference matrix; *NGLDM*, neighborhood grey level dependence matrix.

**Table SR2. Number of CT features remained after each step in Part 1**

**Table SR2.** Number of CT features remained after each step in Part 1

| Feature selection steps | Mor | LI | IS | IH | IVH | GLCM | GLRLM | GLSZM | GLDZM | NGTDM | NGLDM |
| --- | --- | --- | --- | --- | --- | --- | --- | --- | --- | --- | --- |
| Before selection | 29 | 2 | 18 | 23 | 7 | 150 | 96 | 48 | 48 | 15 | 51 |
| Inter-observer reproducibility | 9 | 2 | 11 | 18 | 6 | 119 | 86 | 41 | 39 | 10 | 43 |
| Information evaluation | 9 | 2 | 11 | 18 | 5 | 119 | 86 | 39 | 37 | 10 | 41 |
| Univariate analysis | 8 | 2 | 7 | 14 | 3 | 80 | 64 | 29 | 31 | 8 | 26 |

*Mor*, morphological features; *LI*, local intensity (peak) features; *IS,* intensity-based statistical features; *IH*, intensity histogram features; *IVH*, intensity-volume histogram features; *GLCM*, gray-level co-occurrence matrix; *GLRLM*, gray-level run-length matrix; *GLSZM*, gray level size zone matrix; *GLDZM*, gray level distance zone matrix; *NGTDM*, neighborhood gray tone difference matrix; *NGLDM*, neighborhood grey level dependence matrix.

**Table SR3. Number of PET features remained after each step in Part 2**

**Table SR3.** Number of PET features remained after each step in Part 2

| Feature selection steps | Mor | LI | IS | IH | IVH | GLCM | GLRLM | GLSZM | GLDZM | NGTDM | NGLDM |
| --- | --- | --- | --- | --- | --- | --- | --- | --- | --- | --- | --- |
| Before selection | 12 | 2 | 15 | 15 | 4 | 111 | 91 | 40 | 45 | 9 | 42 |
| Redundancy reduction | 1 | 0 | 0 | 0 | 0 | 1 | 0 | 2 | 1 | 0 | 0 |
| LASSO | 0 | 0 | 0 | 0 | 0 | 1 | 0 | 1 | 1 | 0 | 0 |

*Mor*, morphological features; *LI*, local intensity (peak) features; *IS,* intensity-based statistical features; *IH*, intensity histogram features; *IVH*, intensity-volume histogram features; *GLCM*, gray-level co-occurrence matrix; *GLRLM*, gray-level run-length matrix; *GLSZM*, gray level size zone matrix; *GLDZM*, gray level distance zone matrix; *NGTDM*, neighborhood gray tone difference matrix; *NGLDM*, neighborhood grey level dependence matrix; *LASSO*, least absolute shrinkage and selection operator.

**Table SR4. Number of CT features remained after each step in Part 3**

**Table SR4.** Number of CT features remained after each step in Part 3

|  | Mor and IS features | |  | The remaining CT features | | | | | | | | | | |
| --- | --- | --- | --- | --- | --- | --- | --- | --- | --- | --- | --- | --- | --- | --- |
| Feature selection steps | Mor | IS |  | Mor | LI | IS | IH | IVH | GLCM | GLRLM | GLSZM | GLDZM | NGTDM | NGLDM |
| Before selection | 29 | 18 |  | 8 | 2 | 7 | 14 | 3 | 80 | 64 | 29 | 31 | 8 | 26 |
| Pearson correlation analysis | 7 | 5 |  | n.a. | n.a. | n.a. | n.a. | n.a. | n.a. | n.a. | n.a. | n.a. | n.a. | n.a. |
| Cross-validation in ULR | 1 | 1 |  | n.a. | n.a. | n.a. | n.a. | n.a. | n.a. | n.a. | n.a. | n.a. | n.a. | n.a. |
| Redundancy reduction | n.a. | n.a. |  | 1 | 1 | 1 | 0 | 0 | 0 | 0 | 3 | 1 | 0 | 1 |
| LASSO | n.a. | n.a. |  | 0 | 0 | 1 | 0 | 0 | 0 | 0 | 2 | 1 | 0 | 1 |
| Pearson correlation analysis | 1 | 1 |  | n.a. | n.a. | 1 | n.a. | n.a. | n.a. | n.a. | 1 | 0 | n.a. | 1 |

*Mor*, morphological features; *LI*, local intensity (peak) features; *IS,* intensity-based statistical features; *IH*, intensity histogram features; *IVH*, intensity-volume histogram features; *GLCM*, gray-level co-occurrence matrix; *GLRLM*, gray-level run-length matrix; *GLSZM*, gray level size zone matrix; *GLDZM*, gray level distance zone matrix; *NGTDM*, neighborhood gray tone difference matrix; *NGLDM*, neighborhood grey level dependence matrix; *Cross-validation in ULR*, cross-validation in univariate logistic regression; *LASSO*, least absolute shrinkage and selection operator; *n.a.*, not applicable.

**Table SR5. The Morphological features that** **correlated with MTD**

**Table SR5.** The Morphological features that correlated with MTD

| Morphological features | Pearson Correlation Coefficient | Mean AUC |
| --- | --- | --- |
| morph_volume | 0.81 | 0.78 |
| morph_vol_approx | 0.81 | 0.78 |
| morph_area_mesh | 0.83 | 0.77 |
| morph_av | -0.77 | 0.79 |
| morph_pca_maj_axis | 0.80 | 0.74 |
| morph_pca_min_axis | 0.84 | 0.78 |
| morph_pca_least_axis | 0.79 | 0.77 |

The optimal feature that correlated with MTD was marked with orange

**Table SR6. The** **Intensity-based statistical features that correlated with C/T ratio**

**Table SR6.** The Intensity-based statistical features that correlated with C/T ratio

| Intensity-based statistical features | Pearson Correlation Coefficient | Mean AUC |
| --- | --- | --- |
| stat_mean | 0.87 | 0.79 |
| stat_skew | -0.81 | 0.79 |
| stat_median | 0.89 | 0.81 |
| stat_p90 | 0.82 | 0.80 |
| stat_rms | -0.83 | 0.77 |

The optimal feature that correlated with C/T ratio was marked with orange

**Table SR7. Pearson correlation analysis between LASSO-CT features and morph_av feature or stat_median feature**

**Table SR7.** Pearson correlation analysis between LASSO-CT features and morph_av feature or stat_median feature

| Features | Pearson correlation coefficient with morph_av features | Pearson correlation coefficient with stat_median features |
| --- | --- | --- |
| stat_max | -0.54 | 0.68 |
| szm_hgze_2D | -0.62 | 0.97 |
| szm_lzlge_3D | -0.34 | 0.14 |
| dzm_ldhge_2_5D | -0.83 | 0.67 |
| ngl_hdhge_2_5D | -0.74 | 0.68 |

The removed LASSO-CT features was marked with blue

**Table SR8. The predictive performance of the selected single feature**

**Table SR8.** The predictive performance of the selected single feature

| Image | Features | AUC (95%CI) | |
| --- | --- | --- | --- |
|  |  | Training cohort | Validation cohort |
| PET | cm_energy_3D_avg | 0.79 (0.70-0.88) | 0.65 (0.42-0.77) |
|  | szm_szlge_3D | 0.80 (0.67-0.87) | 0.61 (0.37-0.79) |
|  | dzm_zdnu_2_5D | 0.78 (0.68-0.85) | 0.64 (0.48-0.82) |
| CT | morph_av | 0.80 (0.70-0.88) | 0.63 (0.43-0.85) |
|  | stat_median | 0.82 (0.72-0.89) | 0.57 (0.36-0.77) |
|  | stat_max | 0.82 (0.74-0.89) | 0.70 (0.50-0.84) |
|  | szm_lzlge_3D | 0.79 (0.69-0.88) | 0.57 (0.42-0.72) |
|  | ngl_hdhge_2_5D | 0.81 (0.76-0.88) | 0.59 (0.38-0.73) |

**Table SR9. The predictive performance of radiomics model combined with re-sampling techniques**

**Table SR9.** The predictive performance of radiomics model combined with re-sampling techniques

| Re-sampling techniques | Training cohort (n=130) | | | | | | | | | Validation cohort (n=53) | | | | | | | | |  |
| --- | --- | --- | --- | --- | --- | --- | --- | --- | --- | --- | --- | --- | --- | --- | --- | --- | --- | --- | --- |
|  | | AUC  (95% CI) | G-mean | maP | maR | maF | miP | miR | miF | | AUC  (95% CI) | G-mean | maP | maR | maF | miP | miR | miF | |
| ROS | | 0.84  (0.80-0.88) | 0.81 | 0.78 | 0.88 | 0.83 | 0.87 | 0.75 | 0.80 | | 0.66  (0.49-0.82) | 0.59 | 0.88 | 0.64 | 0.74 | 0.24 | 0.56 | 0.33 | |
| ADASYN | | 0.86  (0.79-0.90) | 0.78 | 0.80 | 0.75 | 0.78 | 0.77 | 0.82 | 0.79 | | 0.64  (0.50-0.79) | 0.58 | 0.87 | 0.61 | 0.72 | 0.23 | 0.56 | 0.32 | |
| SMOTE | | 0.88  (0.85-0.92) | 0.80 | 0.85 | 0.73 | 0.79 | 0.77 | 0.88 | 0.82 | | 0.65  (0.52-0.81) | 0.62 | 0.88 | 0.68 | 0.77 | 0.26 | 0.56 | 0.36 | |
| bSMOTE | | 0.89  (0.85-0.94) | 0.84 | 0.90 | 0.77 | 0.83 | 0.80 | 0.91 | 0.85 | | 0.68  (0.47-0.81) | 0.49 | 0.84 | 0.73 | 0.78 | 0.20 | 0.33 | 0.25 | |
| RUS | | 0.90  (0.76-0.97) | 0.84 | 0.86 | 0.81 | 0.83 | 0.82 | 0.87 | 0.84 | | 0.69  (0.53-0.82) | 0.68 | 0.96 | 0.52 | 0.68 | 0.28 | 0.89 | 0.42 | |
| NM | | 0.91  (0.78-0.97) | 0.82 | 0.90 | 0.73 | 0.81 | 0.77 | 0.92 | 0.84 | | 0.68  (0.55-0.83) | 0.65 | 0.90 | 0.64 | 0.75 | 0.27 | 0.67 | 0.39 | |
| TL | | 0.86  (0.76-0.91) | 0.84 | 0.80 | 0.92 | 0.86 | 0.91 | 0.77 | 0.83 | | 0.67  (0.50-0.80) | 0.62 | 0.88 | 0.68 | 0.77 | 0.26 | 0.56 | 0.36 | |
| ENN | | 0.92  (0.85-0.97) | 0.80 | 0.95 | 0.75 | 0.84 | 0.48 | 0.85 | 0.61 | | 0.71  (0.48-0.83) | 0.51 | 0.85 | 0.80 | 0.82 | 0.25 | 0.33 | 0.29 | |
| SMOTE-TL | | 0.89  (0.83-0.94) | 0.84 | 0.88 | 0.96 | 0.92 | 0.90 | 0.73 | 0.81 | | 0.65  (0.49-0.78) | 0.56 | 0.86 | 0.70 | 0.78 | 0.24 | 0.44 | 0.31 | |
| SMOTE-ENN | | 1.00  (1.00-1.00) | 0.84 | 0.89 | 0.79 | 0.84 | 0.81 | 0.90 | 0.85 | | 0.68  (0.54-0.86) | 0.64 | 0.89 | 0.73 | 0.80 | 0.29 | 0.56 | 0.38 | |

The effective resampling techniques which achieve significantly higher prediction performance than no-resampling were marked with orange. *ROS* random over-sampling, *ADASYN* Adaptive Synthetic, *SMOTE* Synthetic Minority Oversampling Technique, *bSMOTE* Borderline SMOTE, *RUS* Random under-sampling, *NM* Near Miss, *TL* Tomek links, *ENN* Edited Nearest Neighbours, *SMOTE-TL* Over-sampling using SMOTE and cleaning using Tomek links, and *SMOTE-ENN* Over-sampling using SMOTE and cleaning using ENN.

**Table SR10. The predictive performance of traditional model combined with re-sampling techniques**

**Table SR10.** The predictive performance of traditional model combined with re-sampling techniques

| Re-sampling techniques | Training cohort (n=130) | | | | | | | | Validation cohort (n=53) | | | | | | | |
| --- | --- | --- | --- | --- | --- | --- | --- | --- | --- | --- | --- | --- | --- | --- | --- | --- |
|  | AUC  (95% CI) | G-mean | maP | maR | maF | miP | miR | miF | AUC  (95% CI) | G-mean | maP | maR | maF | miP | miR | miF |
| ROS | 0.83  (0.76-0.90) | 0.76 | 0.95 | 0.60 | 0.73 | 0.71 | 0.97 | 0.82 | 0.67  (0.39-0.87) | 0.64 | 0.90 | 0.61 | 0.73 | 0.26 | 0.67 | 0.38 |
| ADASYN | 0.85  (0.79-0.88) | 0.79 | 0.96 | 0.63 | 0.76 | 0.73 | 0.97 | 0.83 | 0.68  (0.42-0.85) | 0.67 | 0.91 | 0.68 | 0.78 | 0.30 | 0.67 | 0.41 |
| SMOTE | 0.85  (0.78-0.89) | 0.82 | 0.84 | 0.78 | 0.81 | 0.79 | 0.86 | 0.82 | 0.66  (0.43-0.81) | 0.65 | 0.89 | 0.75 | 0.81 | 0.31 | 0.56 | 0.40 |
| bSMOTE | 0.86  (0.81-0.89) | 0.83 | 0.88 | 0.78 | 0.83 | 0.80 | 0.89 | 0.85 | 0.66  (0.37-0.91) | 0.64 | 0.89 | 0.73 | 0.80 | 0.29 | 0.56 | 0.38 |
| RUS | 0.88  (0.78-0.95) | 0.83 | 0.84 | 0.81 | 0.82 | 0.81 | 0.85 | 0.83 | 0.65  (0.45-0.81) | 0.64 | 0.89 | 0.73 | 0.80 | 0.29 | 0.56 | 0.38 |
| NM | 0.89  (0.72-0.95) | 0.88 | 0.92 | 0.85 | 0.88 | 0.86 | 0.92 | 0.89 | 0.61  (0.39-0.80) | 0.63 | 0.89 | 0.70 | 0.78 | 0.28 | 0.56 | 0.37 |
| TL | 0.84  (0.72-0.89) | 0.80 | 0.94 | 0.79 | 0.86 | 0.51 | 0.81 | 0.63 | 0.67  (0.43-0.85) | 0.65 | 0.89 | 0.75 | 0.81 | 0.31 | 0.56 | 0.40 |
| ENN | 0.89  (0.79-0.93) | 0.82 | 0.97 | 0.70 | 0.82 | 0.61 | 0.96 | 0.75 | 0.68  (0.36-0.83) | 0.64 | 0.90 | 0.61 | 0.73 | 0.26 | 0.67 | 0.38 |
| SMOTE-TL | 0.85  (0.90-0.92) | 0.82 | 0.88 | 0.75 | 0.81 | 0.78 | 0.90 | 0.84 | 0.66  (0.38-0.83) | 0.64 | 0.89 | 0.73 | 0.80 | 0.29 | 0.56 | 0.38 |
| SMOTE-ENN | 0.97  (0.90-1.0) | 0.94 | 1.00 | 0.89 | 0.94 | 0.94 | 1.00 | 0.97 | 0.63  (0.21-0.80) | 0.63 | 0.89 | 0.70 | 0.78 | 0.28 | 0.56 | 0.37 |

The effective resampling techniques which achieve significantly higher prediction performance than no-resampling were marked with orange. *ROS* random over-sampling, *ADASYN* Adaptive Synthetic, *SMOTE* Synthetic Minority Oversampling Technique, *bSMOTE* Borderline SMOTE, *RUS* Random under-sampling, *NM* Near Miss, *TL* Tomek links, *ENN* Edited Nearest Neighbours, *SMOTE-TL* Over-sampling using SMOTE and cleaning using Tomek links, and *SMOTE-ENN* Over-sampling using SMOTE and cleaning using ENN.

**Table SR11. The predictive performance of combined model combined with re-sampling techniques**

**Table SR11.** The predictive performance of combined model combined with re-sampling techniques

| Re-sampling techniques | Training cohort (n=130) | | | | | | | | Validation cohort (n=53) | | | | | | | |
| --- | --- | --- | --- | --- | --- | --- | --- | --- | --- | --- | --- | --- | --- | --- | --- | --- |
|  | AUC  (95% CI) | G-mean | maP | maR | maF | miP | miR | miF | AUC  (95% CI) | G-mean | maP | maR | maF | miP | miR | miF |
| ROS | 0.87  (0.81-0.91) | 0.81 | 0.95 | 0.68 | 0.79 | 0.75 | 0.96 | 0.84 | 0.71  (0.52-0.93) | 0.74 | 0.94 | 0.70 | 0.81 | 0.35 | 0.78 | 0.48 |
| ADASYN | 0.87  (0.82-0.92) | 0.82 | 0.82 | 0.82 | 0.82 | 0.82 | 0.82 | 0.82 | 0.69  (0.51-0.87) | 0.58 | 0.87 | 0.75 | 0.80 | 0.27 | 0.44 | 0.33 |
| SMOTE | 0.88  (0.91-0.92) | 0.85 | 0.91 | 0.79 | 0.85 | 0.81 | 0.92 | 0.86 | 0.69  (0.37-0.83) | 0.70 | 0.91 | 0.73 | 0.81 | 0.33 | 0.67 | 0.44 |
| bSMOTE | 0.90  (0.84-0.94) | 0.84 | 0.88 | 0.80 | 0.84 | 0.82 | 0.89 | 0.85 | 0.72  (0.52-0.85) | 0.71 | 0.92 | 0.75 | 0.83 | 0.35 | 0.67 | 0.46 |
| RUS | 0.93  (0.82-0.97) | 0.88 | 0.83 | 0.96 | 0.89 | 0.95 | 0.81 | 0.88 | 0.74  (0.53-0.87) | 0.76 | 0.94 | 0.75 | 0.84 | 0.39 | 0.78 | 0.52 |
| NM | 0.91  (0.78-0.98) | 0.86 | 0.83 | 0.92 | 0.87 | 0.91 | 0.81 | 0.86 | 0.70  (0.55-0.88) | 0.66 | 0.89 | 0.77 | 0.83 | 0.33 | 0.56 | 0.42 |
| TL | 0.97  (0.79-0.93) | 0.83 | 0.94 | 0.84 | 0.89 | 0.58 | 0.81 | 0.68 | 0.70  (0.44-0.84) | 0.51 | 0.85 | 0.80 | 0.82 | 0.25 | 0.33 | 0.29 |
| ENN | 0.94  (0.86-0.97) | 0.88 | 0.94 | 0.87 | 0.90 | 0.77 | 0.88 | 0.82 | 0.75  (0.57-0.91) | 0.76 | 0.94 | 0.75 | 0.84 | 0.39 | 0.78 | 0.52 |
| SMOTE-TL | 0.89  (0.84-0.95) | 0.86 | 0.91 | 0.80 | 0.85 | 0.82 | 0.92 | 0.87 | 0.69  (0.50-0.88) | 0.64 | 0.89 | 0.73 | 0.80 | 0.29 | 0.56 | 0.38 |
| SMOTE-ENN | 1.00  (1.00-1.00) | 1.00 | 1.00 | 1.00 | 1.00 | 1.00 | 1.00 | 1.00 | 0.70  (0.48-0.81) | 0.65 | 0.89 | 0.75 | 0.81 | 0.31 | 0.56 | 0.40 |

The effective resampling techniques which achieve significantly higher prediction performance than no-resampling were marked with orange. *ROS* random over-sampling, *ADASYN* Adaptive Synthetic, *SMOTE* Synthetic Minority Oversampling Technique, *bSMOTE* Borderline SMOTE, *RUS* Random under-sampling, *NM* Near Miss, *TL* Tomek links, *ENN* Edited Nearest Neighbours, *SMOTE-TL* Over-sampling using SMOTE and cleaning using Tomek links, and *SMOTE-ENN* Over-sampling using SMOTE and cleaning using ENN.

**Table SR12. The number of patients with or without metastasis in the histologic subtype subgroup in the overall, training and validation cohort**

**Table SR12.** The number of patients with or without metastasis in the histologic subtype subgroup in the overall, training and validation cohort

| **Overall (N=183)** | | | |
| --- | --- | --- | --- |
| **Histologic subtype subgroup** | **LNM** | **Non-metastasis** | ***p* value** |
| Low-grade tumor (N=24) | 0 (0%) | 24 (100%) | 0.004 |
| Intermediate-grade tumor (N=133) | 26 (20%) | 107 (80%) | 0.812 |
| High-grade tumor (N=26) | 9 (34.6%) | 17 (65.4%) | 0.030 |
| **Training cohort (N=130)** | | | |
| **Histologic subtype subgroup** | **LNM** | **Non-metastasis** | ***p* value** |
| Low-grade tumor (N=15) | 0 (0%) | 15 (100%) | 0.040 |
| Intermediate-grade tumor (N=97) | 19 (19.6%) | 78 (80.4%) | 0.840 |
| High-grade tumor (N=18) | 7 (38.9 %) | 11 (61.1%) | 0.031 |
| **Validation cohort (N=53)** | | | |
| **Histologic subtype subgroup** | **LNM** | **Non-metastasis** | ***p* value** |
| Low-grade tumor (N=9) | 0 (0%) | 9 (100%) | 0.136 |
| Intermediate-grade tumor (N=36) | 7 (19.4%) | 29 (80.6%) | 0.487 |
| High-grade tumor (N=8) | 2 (25%) | 6 (75%) | 0.512 |

Categorical variables are in N (%) and analyzed using chi-squared. The low-grade tumor consisted of AIS and MIA, the intermediate-grade comprised of LPA, APA and PPA, while SPA and IMA were considered as the high-grade tumor. *LNM* Lymph node metastasis, *AIS* Adenocarcinoma in situ, *MIA* Minimally invasive adenocarcinomas, *LPA* Lepidic predominant invasive adenocarcinomas, *APA* Acinar predominant adenocarcinomas, *PPA* Papillary predominant adenocarcinomas, *SPA* Solid predominant invasive adenocarcinomas, IMA Invasive mucinous adenocarcinomas

**Figure SR1.** **The distribution of** **Rad-score of the intermediate-grade tumor with or without lymph node metastasis**


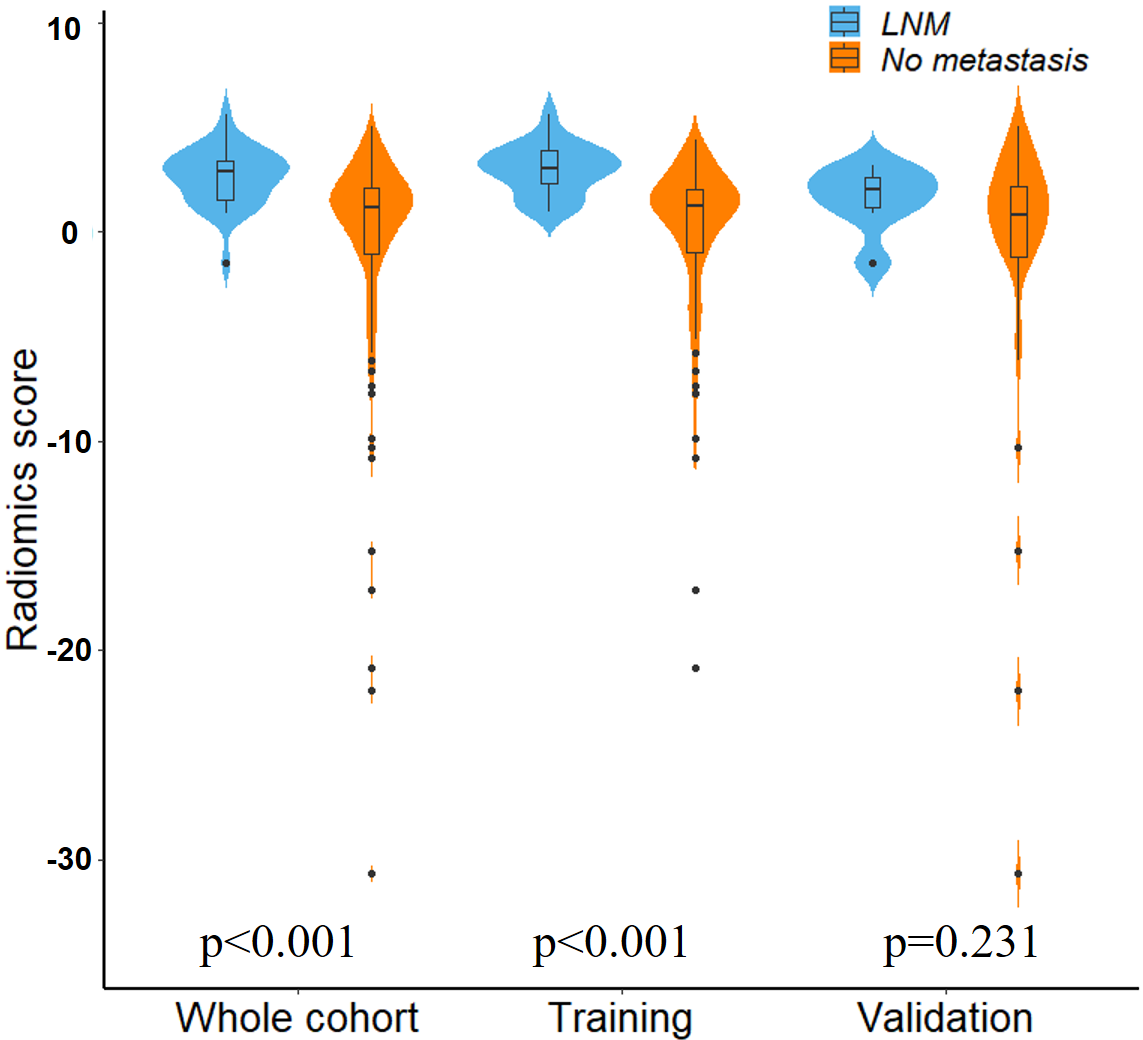


**Figure SR1.** The distribution of Rad-score of the intermediate-grade tumor with or without lymph node metastasis in the whole, training, and validation cohorts. The blue violin plots represent the distribution of radiomics score of the intermediate-grade tumor with LNM in the whole, training and validation cohorts, and the orange violin plots represent the distribution of radiomics score of the intermediate-grade tumor without metastasis in the whole, training and validation cohorts. The longitudinal length of the violin plot indicates the range of the radiomics score, while the transverse width indicates the frequency of the radiomics score. Significant difference of the radiomic score of the intermediate-grade tumor with and without metastasis was found in both the whole and training cohorts (p < 0.001).

**Figure SR2. Confusion matrixes of the radiomics model and traditional model**


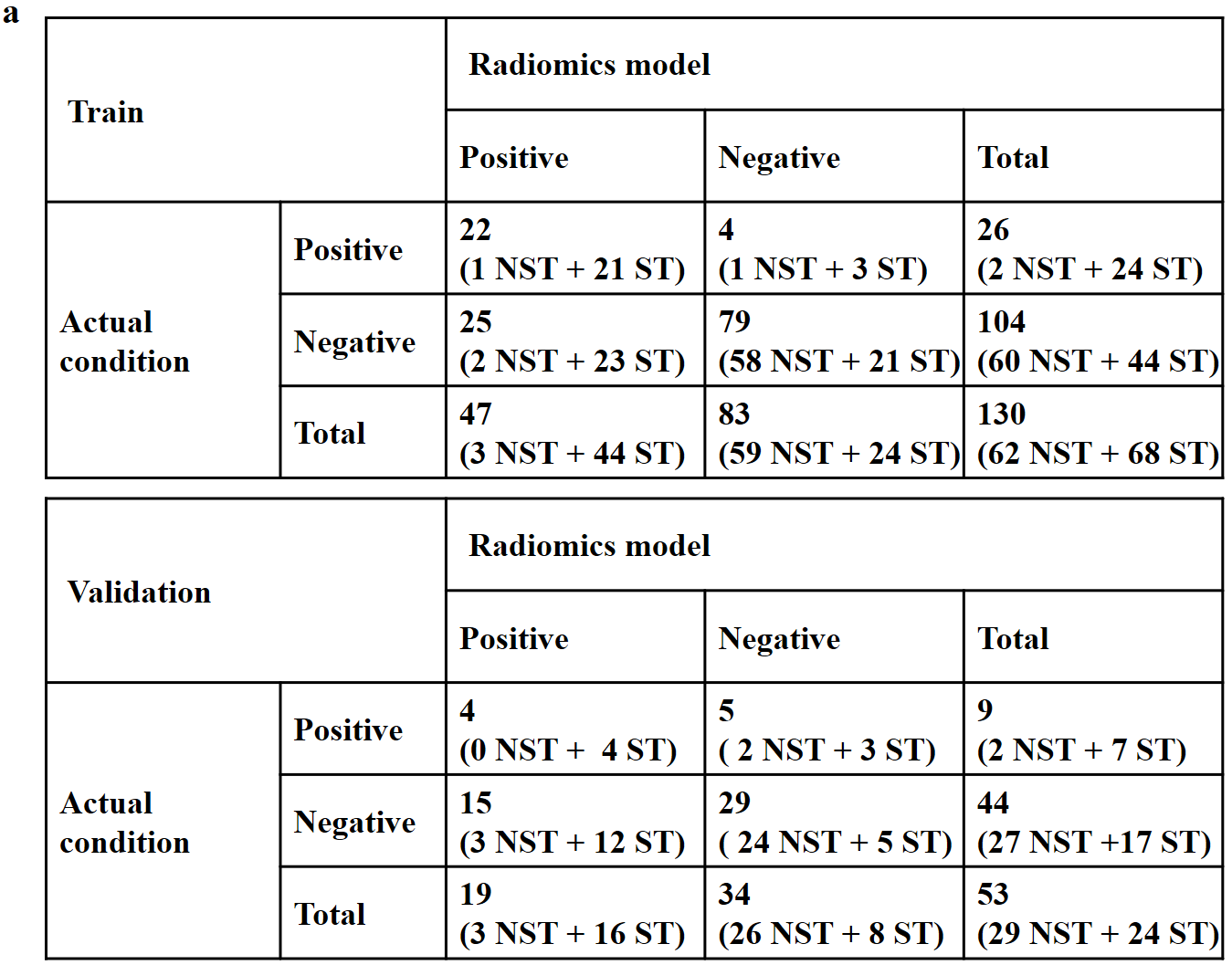


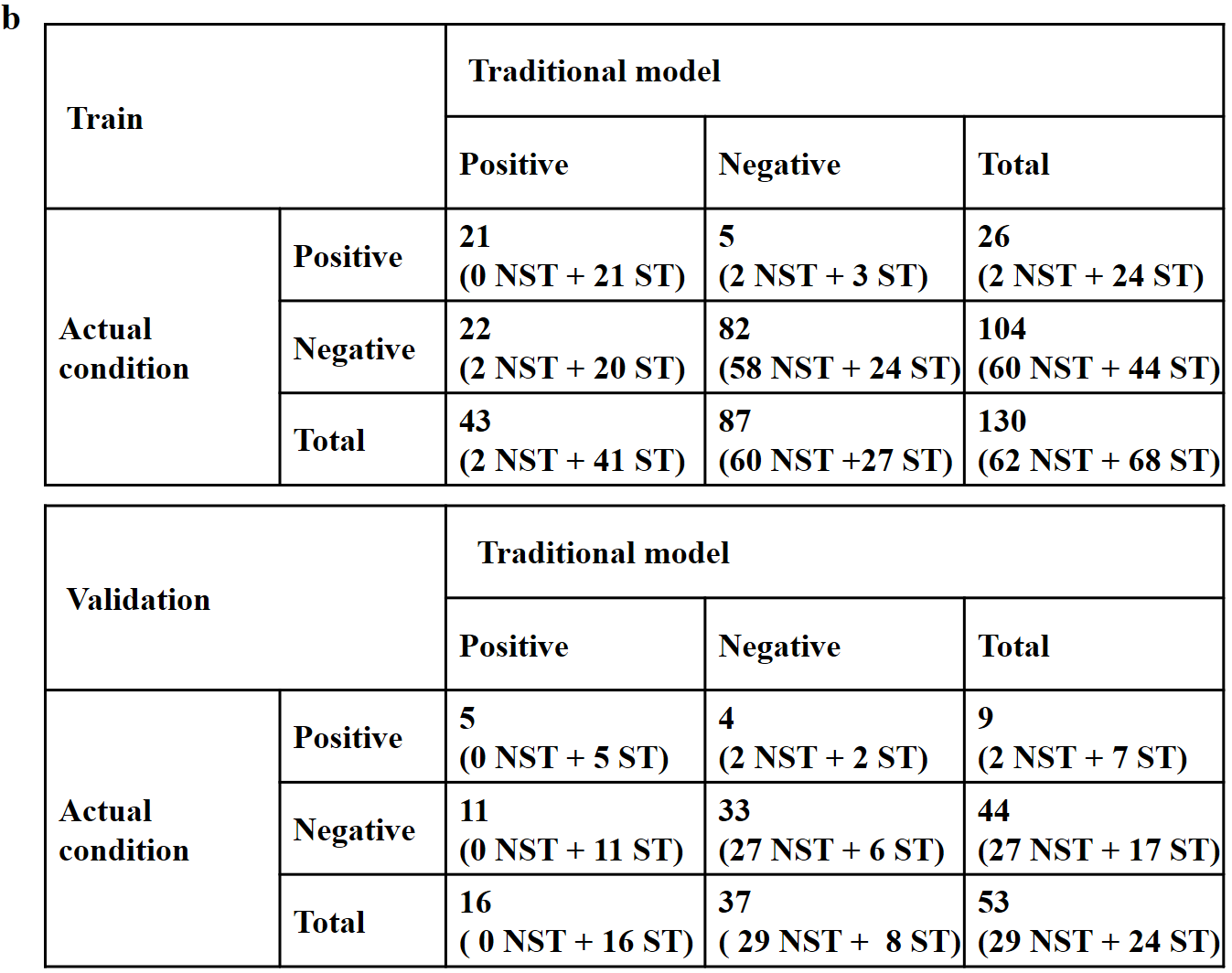


**Figure SR2.** The confusion matrixes of the radiomics model (a) and traditional model (b). The confusion matrixes created based on the prediction performance of (a) radiomics model and (b) traditional model both in the training and validation cohort. The numbers in brackets represent the number of non-solid tumor (NST) and solid tumor (ST) in this category, respectively.

Predicting by the radiomics model, in the training cohort, the number of non-solid tumor that correctly predicted LNM (true positive instances (TP) of NST) was 1 (total=2), and the number of non-solid tumor that correctly predicted non-metastasis (true negative instances (TN) of NST) was 58 (total=60). Thus, the ACC value in NST was 0.95; the number of solid tumor that correctly predicted LNM (TP of ST) was 21 (total= 24), and the number of solid tumor that correctly predicted non-metastasis (TN of ST) was 21 (total=44), the ACC value in the ST was 0.62. In the validation cohort, the number of NST that correctly predicted LNM (TP of NST) was 0 (total=2), and the number of NST that correctly predicted non-metastasis (TN of NST) was 24 (total=27), the ACC value in NST was 0.83; the number of solid tumor that correctly predicted LNM (TP of ST) was 4 (total= 7), and the number of solid tumor that correctly predicted non-metastasis (TN of ST) was 5 (total=17), the ACC value in ST was 0.38.

Predicting by the traditional model, in the training cohort, the number of non-solid tumor that correctly predicted LNM (TP of NST) was 0 (total=2), and the number of non-solid tumor that correctly predicted non-metastasis (TN of NST) was 58 (total=60), the ACC value in NST was 0.94; the number of solid tumor that correctly predicted LNM (TP of ST) was 21 (total= 24), and the number of solid tumor that correctly predicted non-metastasis (TN of ST) was 24 (total=44), the ACC value in ST was 0.66. In the validation cohort, the number of non-solid tumor that correctly predicted LNM (TP of NST) was 0 (total=2), and the number of non-solid tumor that correctly predicted non-metastasis (TN of NST) was 27 (total=27), the ACC value in NST was 0.93; the number of solid tumor that correctly predicted LNM (TP of ST) was 5 (total= 7), and the and the number of solid tumor that correctly predicted non-metastasis (TN of ST) was 6 (total=17), the ACC value in ST was 0.46.

**3. Supplementary Figures**

**Figure S1. The receiver operating characteristic (ROC) curves of the proposed models in the solid tumor subgroup**


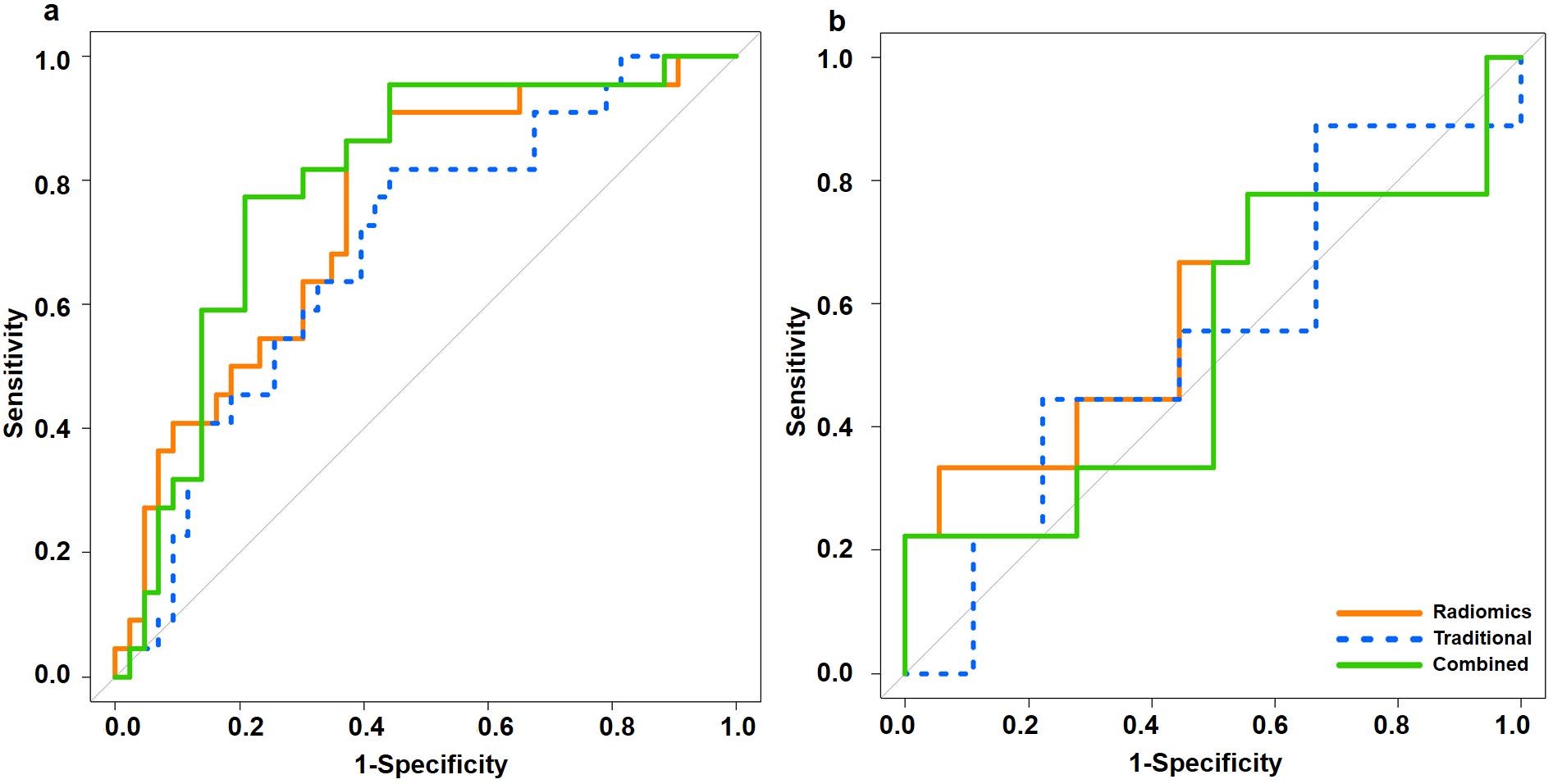


**Figure** **S1.** The receiver operating characteristic (ROC) curves of the proposed models in (a) training and (b) validation cohorts in the solid tumor subgroup. The radiomics model is outperformance for predicting metastasis in the solid tumor subgroup, with the AUC of 0.75 (95% CI 0.61-0.85) and 0.59 (95% CI 0.34-0.90) in the training and validation cohort separately. The combined model improves the prediction performance with respect to the traditional model alone in the training cohort (AUC, 0.79 (95% CI 0.61-0.88) *vs.* 0.69 (95% CI 0.54-0.83); $p$ =0.05),while drooped in the validation cohort (AUC, 0.53 (95% CI 0.25-0.78) *vs.* 0.54 (95% CI 0.32-0.78); $p$ =0.91).

**Figure S2. The F-measure of the majority class (maF) of the proposed models with and without re-sampling techniques**


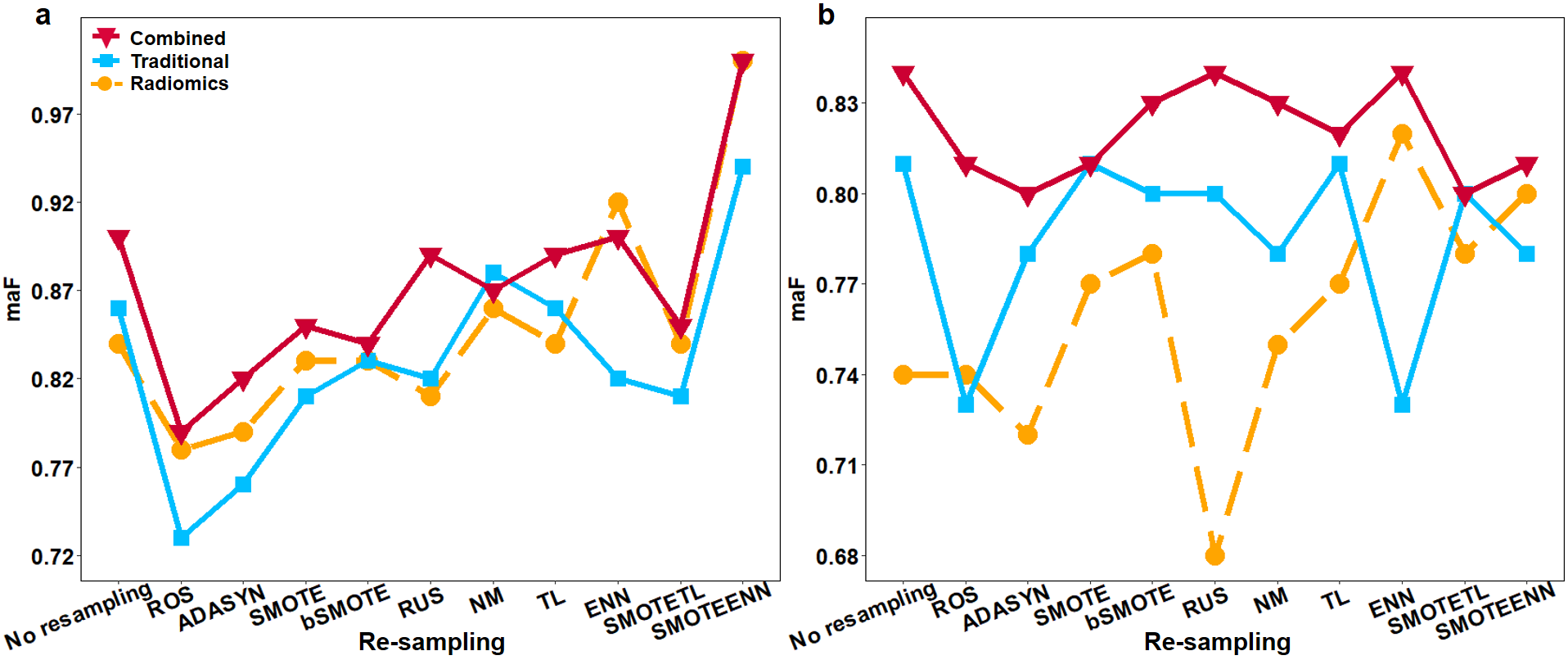


**Figure S2.** The F-measure of the majority class (maF) of the proposed models with and without re-sampling techniques in the (a) training and (b) validation cohorts

**Figure S3. The F-measure of the minority class (miF) of the proposed models with and without re-sampling techniques**


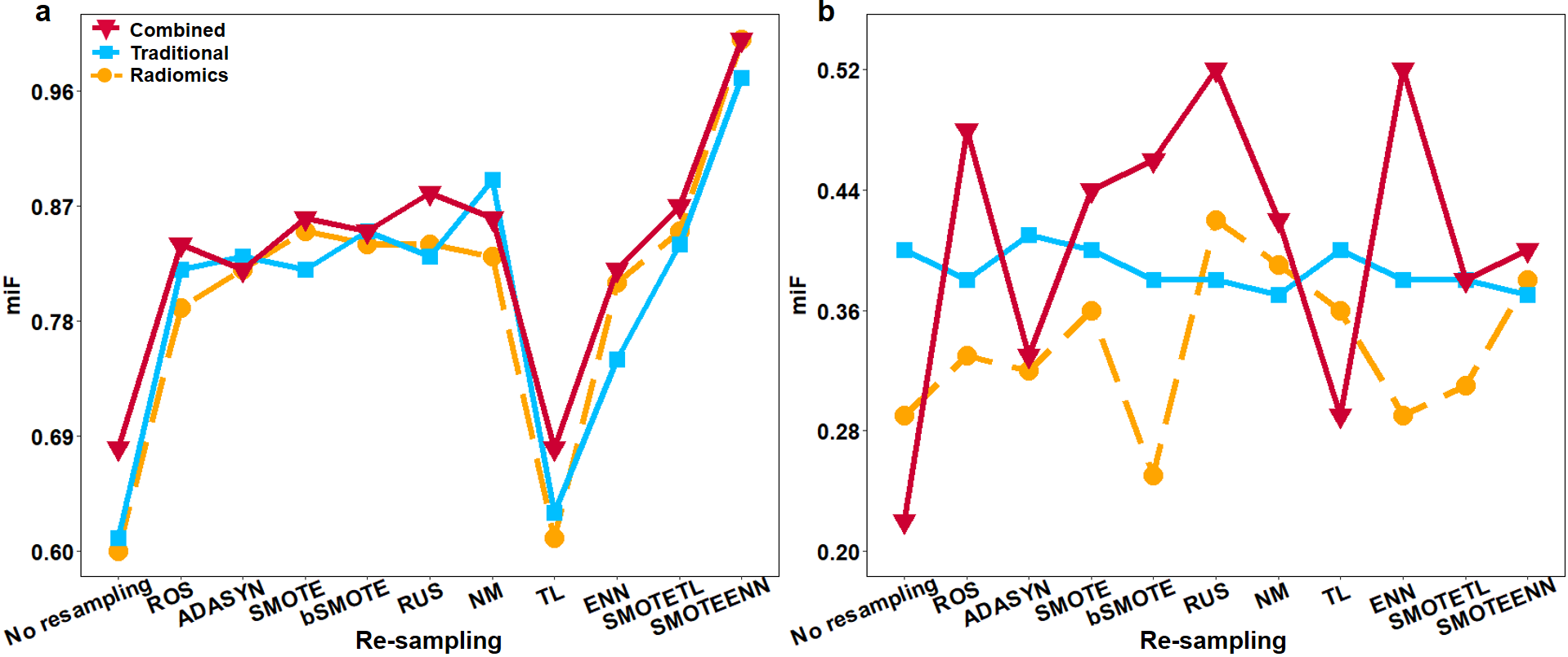


**Figure S3.** The F-measure of the minority class (miF) of the proposed models with and without re-sampling techniques in the (a) training and (b) validation cohorts

**4. Supplementary Tables**

**Tables S1. Data distribution of the training cohort in different re-sampling methods**

**Table S1.** Data distribution of the training cohort in different re-sampling methods

| Dataset | Total number of instances | Number of minority class (LNM) | Number of majority class (non-metastasis) | Imbalance Rate |
| --- | --- | --- | --- | --- |
| No resampling | 130 | 26 | 104 | 1:4 |
| ROS | 208 | 104 | 104 | 1:1.0 |
| ADASYN | 209 | 105 | 104 | 1:0.99 |
| SMOTE | 208 | 104 | 104 | 1:1.0 |
| bSMOTE | 208 | 104 | 104 | 1:1.0 |
| RUS | 52 | 26 | 26 | 1:1.0 |
| NM | 52 | 26 | 26 | 1:1.0 |
| TL | 122 | 26 | 96 | 1:3.69 |
| ENN | 80 | 26 | 54 | 1:2.08 |
| SMOTE-TL | 182 | 91 | 91 | 1:1.0 |
| SMOTE-ENN | 77 | 50 | 27 | 1:0.54 |

*LNM* lymph node metastasis, *ROS* random over-sampling, *ADASYN* Adaptive Synthetic, *SMOTE* Synthetic Minority Oversampling Technique, *bSMOTE* Borderline SMOTE, *RUS* Random under-sampling, *NM* Near Miss, *TL* Tomek links, *ENN* Edited Nearest Neighbours, *SMOTE-TL* Over-sampling using SMOTE and cleaning using Tomek links, and *SMOTE-ENN* Over-sampling using SMOTE and cleaning using ENN.

**Tables S2. The results of univariate logistic regression analysis of the clinicopathological and radiological features**

**Table S2.** The results of univariate logistic regression analysis of the clinicopathological and radiological features

| Characteristics | *p* value | AUC (95% CI) | |
| --- | --- | --- | --- |
|  |  | Training set | validation set |
| Age | 0.03 | 0.61 (0.46-0.69) | 0.33 (0.17-0.67) |
| Gender | 0.12 | 0.57 (0.41-0.70) | 0.55 (0.24-0.77) |
| Histologic subtype | 0.002 | 0.72 (0.60-0.81) | 0.67 (0.51-0.83) |
| EGFR mutation | 0.18 | 0.57 (0.45-0.66) | 0.61 (0.2 9-0.76) |
| MTD | 0.002 | 0.72 (0.61-0.83) | 0.48 (0.29-0.75) |
| C/T ratio | <0.001 | 0.75 (0.68-0.80) | 0.68 (0.37-0.82) |
| MATV | <0.001 | 0.80 (0.73-0.88) | 0.58 (0.39-0.76) |
| TLG | <0.001 | 0.81 (0.72-0.86) | 0.66 (0.46-0.84) |
| SUVpeak | <0.001 | 0.78 (0.69-0.85) | 0.74 (0.56-0.86) |
| SUVmean | <0.001 | 0.78 (0.70-0.85) | 0.73 (0.49-0.85) |
| SUVmax | 0.001 | 0.78 (0.69-0.84) | 0.77 (0.57-0.90) |

The significantly characteristic were marked with orange. *MTD* Maximum tumor diameters, *C/T ratio* Consolidation-to-tumor (C/T) ratio, *AUC* Area under the receiver operating characteristic. * $p$ < 0.05 indicates the significant difference

**Tables S3. Patient characteristics in the solid tumor subgroup**

**Table S3.** Patient characteristics in the solid tumor subgroup

| Characteristic | Subgroup  N= 92 | Training  N= 65 | Validation  N= 27 | $p$ value |
| --- | --- | --- | --- | --- |
| Age (years) | 59.24±9.58 | 60.29±9.79 | 56.70±8.71 | 0.10 |
| Gender |  |  |  | 0.93 |
| Male | 47 (%) | 33 (%) | 14 (%) |  |
| Female | 45 (%) | 32 (%) | 13 (%) |  |
| Overall stage |  |  |  | 0.94 |
| ⅠA1 | 0 (%) | 0 (%) | 0 (%) |  |
| ⅠA2 | 24 (%) | 16 (%) | 8 (%) |  |
| ⅠA3 | 37 (%) | 27 (%) | 10 (%) |  |
| ⅡB | 7 (%) | 6 (%) | 1 (%) |  |
| ⅢA | 23 (%) | 16 (%) | 7 (%) |  |
| IIIB | 1 (%) | 0 (%) | 1 (%) |  |
| Histologic subtype |  |  |  | 0.20 |
| AIS | 1 (%) | 1 (%) | 0 (%) |  |
| MIA | 0 (0%) | 0 (%) | 0 (%) |  |
| LPA | 25 (%) | 21 (%) | 4 (%) |  |
| APA | 38 (%) | 24 (%) | 14 (%) |  |
| PPA | 7 (%) | 5 (%) | 2 (%) |  |
| SPA | 17 (%) | 11 (%) | 6 (%) |  |
| IMA | 4 (%) | 3 (%) | 1 (%) |  |
| EGFR mutation |  |  |  | 0.02 |
| Yes | 52 (%) | 10 (%) | 10 (%) |  |
| No | 40 (%) | 17 (%) | 17 (%) |  |
| MTD (cm) | 2.25±0.49 | 2.27±0.52 | 2.18±0.42 | 0.41 |
| MATV (cm^3^) | 11.36±5.87 | 11.73±6.19 | 10.49±5.00 | 0.42 |
| TLG | 35.41±28.4 | 36.26±29.11 | 33.37±27.6 | 0.82 |
| SUVpeak | 6.70±2.96 | 6.50±3.06 | 7.16±2.71 | 0.42 |
| SUVmean | 2.86±1.06 | 2.81±1.10 | 2.96±0.98 | 0.45 |
| SUVmax | 10.87±4.85 | 10.47±5.15 | 11.83±3.97 | 0.16 |
| LNM |  |  |  | 0.97 |
| Yes | 31 (%) | 22 (%) | 9 (%) |  |
| No | 61 (%) | 43 (%) | 18 (%) |  |

No significant differences were found in overall stage, patient number of metastasis, clinicopathological features and radiological features between the training and validation cohort. Categorical variables are in N (%) and analyzed using chi-squared, while continuous variables are in mean±SD and analyzed using Student’s $t$ test or the Mann-Whitney $U$ test, as appropriate. *LNM* lymph node metastasis, *AIS* Adenocarcinoma in situ, *MIA* Minimally invasive adenocarcinomas, *LPA* Lepidic predominant invasive adenocarcinomas, *APA* Acinar predominant adenocarcinomas, *PPA* Papillary predominant adenocarcinomas, *SPA* Solid predominant invasive adenocarcinomas, *IMA* Invasive mucinous adenocarcinomas, *MTD* Maximum tumor diameters, *C/T ratio* consolidation-to-tumor, *MATV*, metabolically active tumor volume, *TLG* total lesion glycolysis. * $p$ < 0.05 indicates the significant difference

**Tables S4. Comparing the predictive ability between MTD and morph_av radiomics feature**

**Table S4.** Comparing the predictive ability between MTD and morph_av radiomics feature

| Features |  |  | Majority class (Non-metastasis) | | | Minority class (LNM) | | |
| --- | --- | --- | --- | --- | --- | --- | --- | --- |
|  | AUC | G-mean | Precision | Recall | F-measure | Precision | Recall | F-measure |
| MTD | 0.65±0.08 | 0.58±0.07 | 0.83±0.05 | 0.56±0.11 | 0.66±0.08 | 0.33±0.06 | 0.63±0.16 | 0.42±0.07 |
| morph_av | 0.75±0.06 | 0.70±0.06 | 0.89±0.05 | 0.65±0.08 | 0.75±0.05 | 0.42±0.07 | 0.75±0.11 | 0.54±0.07 |
| $p$ value | <0.001 | <0.001 | <0.001 | <0.001 | <0.001 | <0.001 | <0.001 | <0.001 |

The results (mean±SD) calculated form 100 times Monte Carlo cross-validation using univariate logistic regression. *LNM* Lymph node metastasis, *MTD* Maximum tumor diameters, *morph_av* Surface to volume ratio, *AUC* Area under the receiver operating characteristic, *G-mean* Geometric mean score * $p$ < 0.05 indicates the significant difference

**Tables S5. Comparing the predictive ability between C/T ratio and stat_median radiomics feature**

**Table S5.** Comparing the predictive ability between C/T ratio and stat_median radiomics feature

| Features |  |  | Majority class (Non-metastasis) | | | Minority class (LNM) | | |
| --- | --- | --- | --- | --- | --- | --- | --- | --- |
|  | AUC | G-mean | Precision | Recall | F-measure | Precision | Recall | F-measure |
| C/T ratio | 0.73±0.05 | 0.68±0.05 | 0.90±0.05 | 0.58±0.07 | 0.70±0.05 | 0.39±0.04 | 0.81±0.10 | 0.53±0.05 |
| stat_median | 0.75±0.06 | 0.67±0.07 | 0.87±0.05 | 0.66±0.10 | 0.75±0.07 | 0.41±0.07 | 0.69±0.14 | 0.51±0.07 |
| $p$ value | 0.014 | 0.165 | <0.001 | <0.001 | <0.001 | 0.0.74 | <0.001 | 0.714 |

The results (mean±SD) calculated form 100 times Monte Carlo cross-validation using univariate logistic regression. *LNM* Lymph node metastasis, *C/T ratio* Consolidation-to-tumor (C/T) ratio, *stat_median* Intensity-based statistical median feature, *AUC* Area under the receiver operating characteristic, *G-mean* Geometric mean score * $p$ < 0.05 indicates the significant difference

**Tables S6.** **Comparing the predictive performance of different order between feature selection and re-sampling data in the machine learning pipeline in radiomics analysis**

**Table. S6** Comparing the predictive performance of different order between feature selection and re-sampling data in the machine learning pipeline in radiomics analysis

| Sequences |  |  | Majority class (Non-metastasis) | | | Minority class (LNM) | | |
| --- | --- | --- | --- | --- | --- | --- | --- | --- |
|  | AUC | G-mean | Precision | Recall | F-measure | Precision | Recall | F-measure |
| feature selection before resampling data | 0.76±0.06 | 0.67±0.08 | 0.91±0.04 | 0.67±0.10 | 0.76±0.07 | 0.33±0.07 | 0.69±0.17 | 0.43±0.08 |
| resampling data before selecting features | 0.70±0.07 | 0.62±0.10 | 0.88±0.04 | 0.67±0.10 | 0.75±0.07 | 0.29±0.07 | 0.60±0.19 | 0.39±0.09 |
| $p$ value | <0.001 | <0.001 | <0.001 | 0.678 | 0.014 | <0.001 | <0.001 | <0.001 |

*AUC* Area under the receiver operating characteristic, *G-mean* Geometric mean score, *LNM* Lymph node metastasis,

* $p$ < 0.05 indicates the significant difference

**5. References**

1. Travis WD, Brambilla E, Noguchi M, Nicholson AG, Geisinger KR, Yatabe Y, et al. International association for the study of lung cancer/american thoracic society/european respiratory society international multidisciplinary classification of lung adenocarcinoma. *J Thorac Oncol* (2011) 6:244-285. doi: 10.1097/JTO.0b013e318206a221
2. Boellaard R, Delgado-Bolton R, Oyen WJ, Giammarile F, Tatsch K, Eschner W, et al. FDG PET/CT: EANM procedure guidelines for tumour imaging: version 2.0. *Eur J Nucl Med Mol Imaging* (2015) 42:328–354. doi: 10.1007/s00259-014-2961-x
3. Ashrafinia S, Dalaie P, Yan R, Huang P, Pomper M, Schindler T, et al. Application of texture and radiomics analysis to clinical myocardial perfusion SPECT imaging. *J Nucl Med* (2018) 59:94
4. Ashrafinia S (2019) Quantitative nuclear medicine imaging using advanced image reconstruction and radiomics. [dissertation/Ph.D. thesis] Johns Hopkins University
5. Zwanenburg A, Leger S, Vallières M, Löck S. Image biomarker standardisation initiative. arXiv [preprint]. (2016) arXiv:1612.07003
6. Zwanenburg A, Vallieres M, Abdalah MA, Aerts H, Andrearczyk V, Apte A, et al. The Image Biomarker Standardization Initiative: Standardized Quantitative Radiomics for High-Throughput Image-based Phenotyping. *Radiology* (2020) 295:328-338. doi:
7. Lu L, Lv W, Jiang J, Ma J, Feng Q, Rahmim A, et al. Robustness of Radiomic Features in [(11)C]Choline and [(18)F]FDG PET/CT Imaging of Nasopharyngeal Carcinoma: Impact of Segmentation and Discretization. *Mol Imaging Biol* (2016) 18:935-945. doi: 10.1007/s11307-016-0973-6
8. Haibo He, Yang Bai, E. A. Garcia and Shutao Li, "ADASYN: Adaptive synthetic sampling approach for imbalanced learning," 2008 IEEE International Joint Conference on Neural Networks (IEEE World Congress on Computational Intelligence) (2008) pp:1322-1328. doi: 10.1109/IJCNN.2008.4633969.
9. N. V. Chawla, K. W. Bowyer, L. O. Hall, W. P. SMOTE: synthetic minority over-sampling technique. *JAIR* (2002) 16: 321-357. doi: 10.1613/jair.953
10. Han H., Wang WY., Mao BH. “Borderline-SMOTE: A New Over-Sampling Method in Imbalanced Data Sets Learning,”. In: Huang DS., Zhang XP., Huang GB, etitor. Advances in Intelligent Computing. ICIC 2005. Springer, Berlin, Heidelberg (2005). Lecture Notes in Computer Science, vol 3644. doi:10.1007/11538059_91
11. Mani, Inderjeet, I. Zhang. kNN approach to unbalanced data distributions: a case study involving information extraction [Conference presentation]. Proceedings of workshop on learning from imbalanced datasets, ICML United States. (2003) Vol. 126.
12. Two Modifications of CNN. *IEEE Transactions on Systems Man and Communications* (1976) 6(1):769-772. doi: 10.1109/TSMC.1976.4309452
13. Wilson, Dennis L. Asymptotic properties of nearest neighbor rules using edited data. *IEEE Transactions on Systems, Man, and Cybernetics* (1972) 2(3):408-421 doi: 10.1109/TSMC.1972.4309137
14. Batista GEAPA, Prati RC, Monard MC. A study of the behavior of several methods for balancing machine learning training data. *SIGKDD Ex*plor Newsl (2004) 6:20–29. doi: 10.1145/1007730.1007735
15. Batista, Gustavo EAPA, Ana LC Bazzan, Maria Carolina Monard. Balancing Training Data for Automated Annotation of Keywords: a Case Study. *WOB* (2003), pages 10–18
16. Tibshirani R. Regression shrinkage and selection via the Lasso: a retrospective. *J R Stat Soc Series B Stat Methodology* (2011) 73:273–282. doi: 10.1111/j.1467-9868.2011.00771.x
